# Artificial intelligence, explainable deep learning, and generative models for osteoporosis and bone disease prediction: a systematic review and future research roadmap

**DOI:** 10.3389/frai.2026.1930437

**Published:** 2026-10-02

**Authors:** M. Raja, Avulapalli Jayaram Reddy

**Affiliations:** School of Computer Science Engineering and Information Systems, Vellore Institute of Technology, Vellore, Tamil Nadu, India

**Keywords:** explainable artificial intelligence (XAI), federated learning, generative artificial intelligence, multimodal learning, osteoporosis

## Abstract

Osteoporosis is a major global health burden, with considerable morbidity, mortality, and healthcare costs. Artificial intelligence (AI) has opened new avenues for osteoporosis screening, bone mineral density quantification, fracture-risk prediction, and clinical decision support, but progress remains fragmented across machine learning, deep learning, explainable AI (XAI), generative models, and multimodal learning. This systematic review brings these strands together and identifies priority research needs for AI-based osteoporosis prediction. Following the PRISMA 2020 statement, studies were identified from Scopus and Google Scholar, published between January 2020 and the final search date of 30 June 2026, with two reviewers screening independently and resolving disagreements by consensus. Design-related risk-of-bias indicators, such as retrospective vs. prospective design, validation strategy, and reference standard, were recorded for every study; a full PROBAST, QUADAS-2, and Newcastle-Ottawa Scale appraisal is planned but not yet complete. Fifty-eight studies met the inclusion criteria and were synthesized narratively and descriptively, without formal meta-analysis, given substantial heterogeneity. Findings spanned five themes: classical machine learning, deep learning for imaging, explainable AI, generative models, and multimodal learning. Deep learning achieved strong predictive accuracy (AUC 0.82–0.96), XAI improved clinical interpretability, generative models helped offset data scarcity, and multimodal approaches boosted performance by combining imaging and clinical data. Key gaps remain, including limited external validation, heavy reliance on retrospective data, poor generalisability, and limited regulatory readiness. Building on these findings, this review proposes MXGNet, a multimodal, explainable, generative framework incorporating privacy-preserving federated learning, intended to guide development of robust, interpretable, clinically deployable AI tools for osteoporosis prediction.

## 1 Introduction

Osteoporosis is a chronic disease characterized by reduced bone mass and deterioration of bone microarchitecture, resulting in increased bone fragility and fracture susceptibility (Yi et al., 2026; Kim and Kong, 2026). With over 200 million individuals affected worldwide, osteoporosis represents one of the most important public health challenges of an aging world (Wang Q. et al., 2026). The disease is estimated to cause more than 8.9 million fractures annually, with one in three women and one in five men over 50 expected to sustain a fragility fracture during their lifetime (Zhang et al., 2026). Projections indicate that over 50% of global hip fractures will occur in Asia by 2050 as demographic transitions accelerate (Chen et al., 2026). Osteoporosis is a clinical and financial burden. The mortality rate after hip fracture is about 20% in the first year, and patients who survive often are permanently disabled and have a significantly impaired quality of life (Wáng, 2026). Annual US healthcare costs associated with fractures are estimated to exceed $25.3 billion by the year 2025 (Padmanabha and Pai, 2025). Following from the guidelines, less than 20% of older women are considered high risk and less than 5% of high-risk men are actually scanned using DXA in clinical practice (Zhang et al., 2026), and over 50% of all fragility fractures occur in people with osteopenic or even normal BMD, indicating stark limitations of *T*-score thresholds alone as risk stratifiers (Serpa et al., 2026). Among these techniques, Dual-energy X-ray Absorptiometry (DXA) is the current standard for osteoporosis diagnosis, but its application in population screening is constrained by the factors of cost, portability, radiation exposure, and the requirement of a specially trained professional (Shariatnia et al., 2026). These are significant impediments to care, contributing to a large “osteoporosis care gap,” especially in resource-poor settings and among marginalized populations such as men, younger adults, and non-White individuals (Chen et al., 2026; Jayarajah et al., 2026). This discrepancy highlights an urgent need for scalable, affordable, and fair diagnostic advancement. Artificial Intelligence (AI) includes machine learning (ML), deep learning (DL), and generative modeling, which is a disruptive approach in the domain of medical imaging and clinical decision support. In the context of osteoporosis and bone disease, AI methods have the potential to: (1) facilitate opportunistic screening using routinely acquired imaging at no additional cost or radiation (Serpa et al., 2026; Jayarajah et al., 2026); (2) combine diverse clinical, laboratory, and imaging information for enhanced risk stratification (Liu et al., 2022); (3) provide the basis for transparent, explainable predictions that clinicians are willing to trust and treat (Alili et al., 2026); and (4) address the challenge of data scarcity via synthetic data generation (Padmanabha and Pai, 2025). Nonetheless, the literature is disparate with significant variability in study design, imaging modality, population, and evaluation scheme.

This systematic review addresses four interconnected research questions:

- (RQ1)—What ML and DL architectures have been applied to BMD prediction and osteoporosis risk classification, and what performance metrics have been reported?
- (RQ2)—How have explainable AI (XAI) techniques been implemented to make bone disease AI models interpretable and clinically trustworthy?
- (RQ3)—What role do generative models, including GANs and diffusion models, play in augmenting bone imaging data and enabling novel diagnostic frameworks?
- (RQ4)—What are the critical gaps, methodological limitations, and priority directions for future research in AI-driven osteoporosis management?

The contribution of this review is that it consolidates all three components of modern AI-based supervised prediction, explainability, and generative modeling in a single bone disease context and provides a structured future research road map based on evidence synthesis. To guide the reader, Figure 1 illustrates a taxonomy of the AI techniques reviewed in this area, which are classified into five major themes (classical machine learning, deep learning for imaging, explainable AI, generative models, and multimodal fusion) as well as the downstream clinical tasks each of them targets.

**Figure 1:**
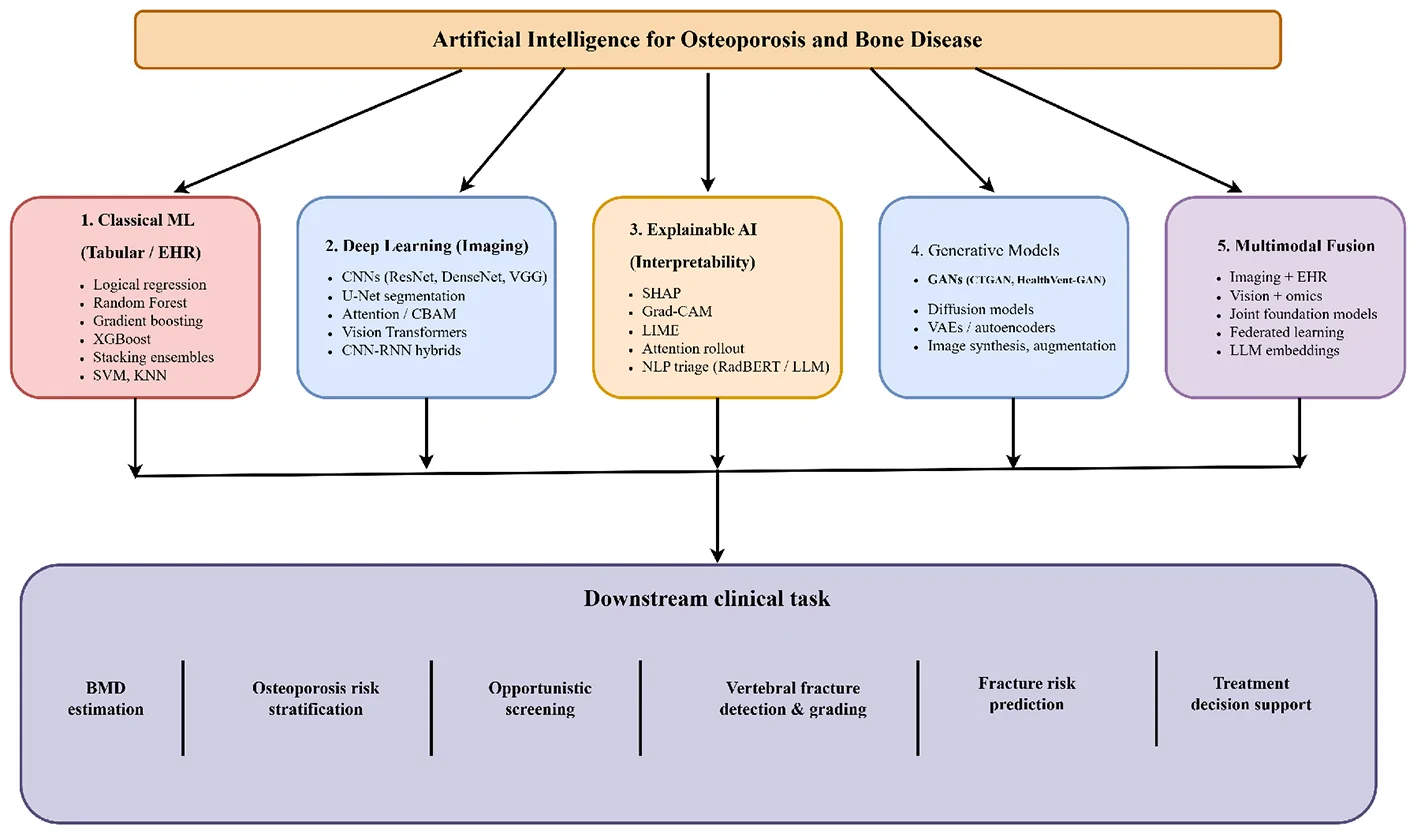
Taxonomy of AI methods for osteoporosis and bone disease prediction.

## 2 Methods

### 2.1 Search strategy

This review was prepared following the PRISMA 2020 statement guidelines. We conducted a literature search through Scopus and Google Scholar to identify articles from January 2020 to June 2026. The search strategy was organized according to six thematic areas: (i) osteoporosis and AI, (ii) osteoporotic imaging, (iii) multimodal learning, (iv) explainable AI (XAI), (v) GANs and synthetic data production, and (vi) diffusion models.

Keywords were combined with the Boolean operators: “osteoporosis” AND “artificial intelligence,” “osteoporosis” AND “machine learning,” “osteoporosis” AND “deep learning,” “bone mineral density” AND “deep learning,” “osteoporosis” AND “X-ray” AND “deep learning,” “osteoporosis” AND “CT” AND “deep learning,” “osteoporosis” AND “MRI” AND “deep learning,” “osteoporosis” AND “multimodal,” “osteoporosis” AND “SHAP,” “osteoporosis” AND “Grad-CAM,” “osteoporosis” AND “GAN,” “osteoporosis” AND “data augmentation,” and “osteoporosis” AND “diffusion model.” Other studies on vertebral fracture prediction, hip fracture prediction, detection of osteopenia, estimation of bone density, musculoskeletal imaging, and orthopedic deep learning were also reviewed due to their relevance to the topic of osteoporosis risk evaluation.

To improve the transparency and reproducibility of the search, the complete database-specific search strings are reported below. For Scopus: TITLE-ABS-KEY ((“osteoporosis” OR “osteopenia” OR “bone mineral density”) AND (“artificial intelligence” OR “machine learning” OR “deep learning” OR “neural network” OR “SHAP” OR “Grad-CAM” OR “explainable AI” OR “generative adversarial network” OR “GAN” OR “diffusion model” OR “multimodal”)) AND *PUBYEAR*>2019 AND PUBYEAR < 2027 AND (LIMIT-TO(LANGUAGE, “English”)). An analogous string, adapted to the platform's syntax, was applied in Google Scholar, restricted to the same date range (1 January 2020–30 June 2026) and to English-language records.

The final literature search across both databases was completed on 30 June 2026. Only English-language, peer-reviewed journal articles, some review articles and conference papers meeting the eligibility criteria in Section 2.2 were considered; non-peer-reviewed preprints (e.g., medRxiv, arXiv, bioRxiv) were excluded *a priori*. For included references carrying a 2026 publication year, eligibility was based on the article having a final, citable digital object identifier (DOI) as a published “online-first”/early-access version of record in a peer-reviewed journal at the time of the search; conference abstracts without accompanying full-text data were excluded regardless of year, as described in Section 2.2 and reported in the PRISMA flow diagram (Figure 2). No study protocol was pre-registered for this review, which we acknowledge as a limitation in Section 7.

**Figure 2:**
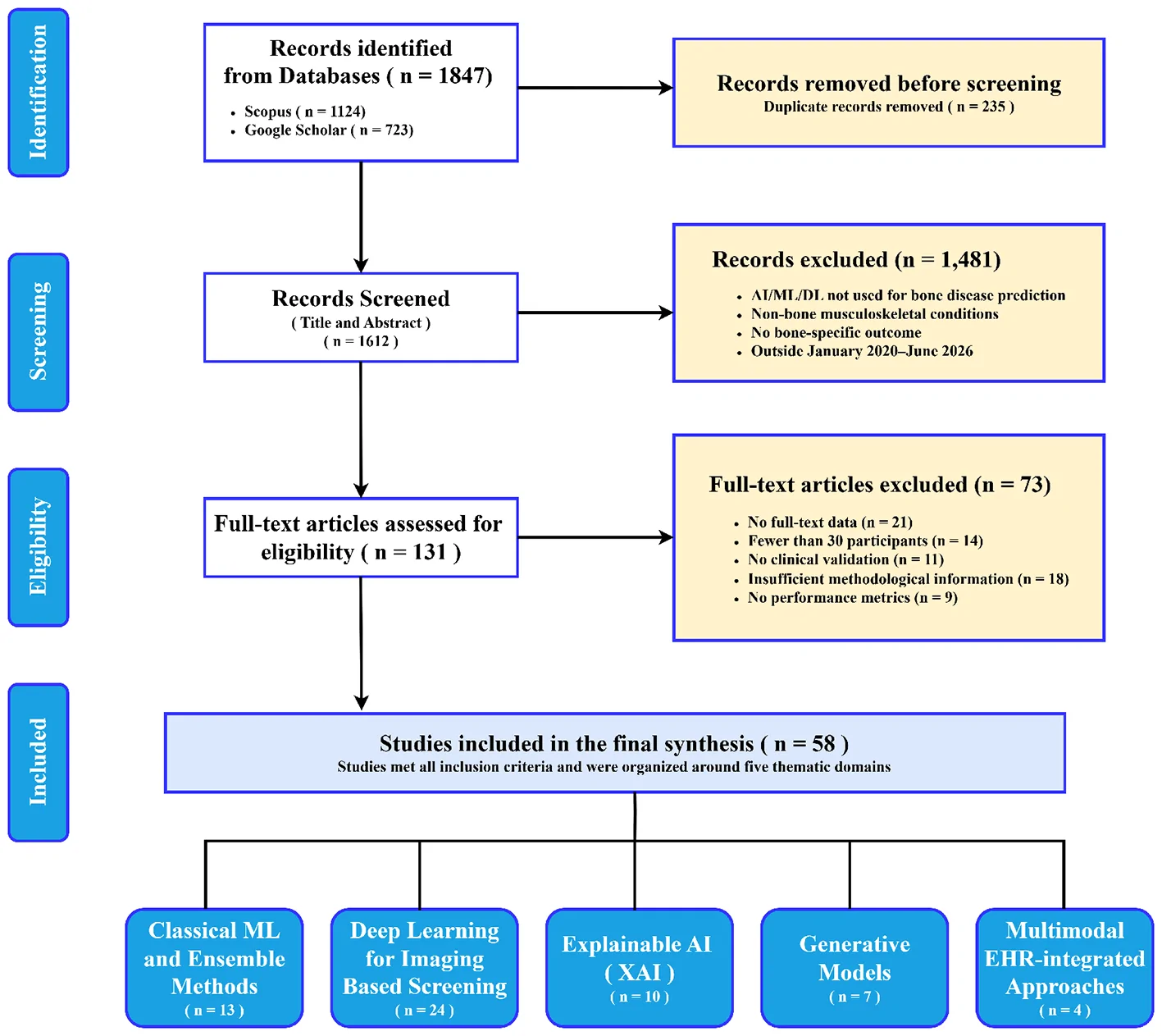
PRISMA 2020 flow diagram.

### 2.2 Inclusion and exclusion criteria

We considered studies that employed Artificial Intelligence (AI), machine learning (ML), deep learning (DL), explainable AI (XAI), generative models, or multimodal learning approaches for the diagnosis of osteoporosis.

Included were: studies utilizing AI/ML/DL methods for osteoporosis screening, bone mineral density (BMD) assessment, fracture risk prediction, or treatment planning; studies reporting quantitative performance metrics (AUC, accuracy, sensitivity, specificity, and F1-score), and peer-reviewed primary studies or high-quality review articles published in English.

Excluded were: conference abstracts without full text, studies with fewer than 30 participants, pure simulation studies with no clinical data validation, and studies applying AI to non-bone musculoskeletal conditions without bone-specific endpoints.

Duplicate records were identified in a two-step process to improve reproducibility. All records exported from Scopus and Google Scholar were first imported into Mendeley Reference Manager, where automated de-duplication was performed using matching algorithms based on DOI, title similarity, and author/year combinations. The automatically flagged pairs, together with any records with high title similarity that were not automatically merged, were then manually reviewed by both authors within Mendeley to confirm true duplication before removal. This combined automated-plus-manual process yielded the 235 duplicate records removed prior to title/abstract screening, as reported in Section 3.1.

Inter-reviewer agreement was not formally quantified using a statistic such as Cohen's kappa in the original conduct of this review. Screening was conducted independently by two reviewers at both the title/abstract and full-text stages, and all discrepancies were resolved through discussion and consensus, with a third party available for adjudication if consensus could not be reached (none was required). We acknowledge the absence of a formal concordance statistic as a methodological limitation in Section 7 and recommend that future updates of this review log reviewer-level decisions prospectively (e.g., using Rayyan or Covidence) to enable this calculation.

For clarity, Mendeley reference manager was the only software tool used in the conduct of this review, applied solely for reference management and de-duplication as described above. No statistical software or concordance-calculation tool was used at any stage of study screening, data extraction, or quality appraisal; where a quantitative statistic (such as Cohen's kappa) or a completed instrument-based appraisal is not reported in this manuscript, this reflects that no such tool was applied, rather than an omission of results that exist elsewhere.

### 2.3 Data extraction and quality assessment

Data extraction was independently performed by two authors using a predefined form for this review. Studied variables were design of the study, features of the participants, applied imaging modalities, employed artificial intelligence techniques, strategies of training and validation, performance metrics, methods for explainable AI (XAI), and the application of generative models. Disagreements during extraction were discussed, and consensus was reached. Each included study was reviewed systematically for methodological structure, dataset size and composition, sampling method, approach to performance evaluation, and key findings to allow for an informative synthesis of the current state of the art.

A formal, instrument-based quality (risk-of-bias) appraisal is planned for this review, using study-design-appropriate validated tools: the Prediction model Risk Of Bias ASsessment Tool (PROBAST) for studies developing or validating an AI/ML prediction model (the majority of the included evidence); QUADAS-2 for studies primarily reporting diagnostic accuracy against a reference standard (e.g., DXA-based *T*-score); and the Newcastle-Ottawa Scale (NOS) for observational cohort studies without a formal predictive-model component. A full domain-by-domain appraisal of all 58 included studies against these instruments has not yet been completed and is not reported as having taken place in this version of the manuscript; we regard this as a methodological limitation, discussed further in Section 7, rather than describe an appraisal that has not occurred. In its place, Section 3.1.2 reports study-design-level indicators captured during data extraction for every included study—study design (retrospective vs. prospective), validation strategy (internal-only, temporal, or external/multicentre), and reference standard used—which are directly relevant to risk of bias and are used, together with the intended PROBAST/QUADAS-2/NOS domains, as a transparent basis for qualifying the strength of the evidence in Sections 4 and 5. Completing the full instrument-based appraisal is identified as a priority next step in Section 7.

### 2.4 Evidence synthesis framework

A thematic synthesis approach was adopted. Five thematic domains emerged from the evidence:

- Classical ML and ensemble methods for clinical risk prediction.
- Deep learning for opportunistic imaging-based screening.
- XAI integration for clinical trust and interpretability.
- Generative models for data augmentation and synthetic image synthesis.
- Multimodal fusion and EHR-integrated approaches.

Within each theme, Quantitative performance data were tabulated, and qualitative synthesis was performed to identify patterns, inconsistencies, and gaps. Each thematic subsection in Section 3 closes with an explicit cross-study synthesis paragraph that draws out shared trends, methodological differences, and gaps across the studies in that theme, rather than presenting only study-by-study descriptions.

### 2.5 Bibliometric and quantitative synthesis methods

A descriptive literature review was performed to categorize the included studies by country, study objective, year of publication, and publication source. In addition, narrative summarization results of AI models were compared with the results of the most commonly reported metrics, which are AUC, accuracy, sensitivity, specificity, precision, recall, and F1-score. To our knowledge, a classic meta-analysis was not performed for the included studies. However, this was not feasible for us given the very large heterogeneity in imaging methodologies, datasets, definitions of outcomes, and modes of validation among the included studies. Instead, the reported performance measures were synthesized descriptively to provide a comprehensive overview of recent developments in AI-based osteoporosis prediction and diagnosis.

Because the performance metrics reported are derived from studies with varying sizes of study populations, validation schemes (internal vs. external/temporal), reference standards, and outcome definitions, readers should not view differences in point estimates (e.g., an AUC of 0.96 vs. 0.89) as proof that one model or modality is absolutely better than another. This caveat is also explicitly stated in Section 3.7 and Section 4.1 whenever comparative statements are presented.

## 3 Results

### 3.1 Study selection and characteristics

The process of study selection followed the PRISMA 2020 statement and is shown in Figure 2. The six thematic query clusters were used to conduct three searches on each of them in two databases, Scopus and Google Scholar, with a total of 1,847 records retrieved (Scopus: *n* = 1, 124; Google Scholar: *n* = 723). After automated and manual deduplication, 235 duplicates were removed, resulting in 1,612 unique records for title and abstract screening. The pre-specified inclusion and exclusion criteria were applied by two independent reviewers, and at this point, any disagreement was resolved by consensus. Almost all of the records (*n* = 1, 481; 91.9%) were excluded at the title and abstract screening stage, because most of them either (1) did not use AI, machine learning (ML), or deep learning (DL) in predicting bone diseases, (2) examined other musculoskeletal issues (similar health issues but not related to bone), or they did not have bone- specific end points, or (3) were published outside the time window of January 2020–June 2026.

After the initial screening, 131 records underwent a full-text evaluation to determine their eligibility. During this phase, 73 articles were eliminated due to various reasons: 21 were conference abstracts without full-text data, 14 had fewer than 30 participants, 11 were solely simulation or phantom studies without clinical data validation, 18 focused exclusively on non-bone musculoskeletal applications of AI, and nine did not present any measurable performance indicators. Upon thorough review and confirmation of eligibility through qualitative synthesis, 58 studies were finally selected to be part of this systematic review. The detailed PRISMA flowchart, showing all exclusions at each step, is displayed in Figure 2.

There were 1,847 results in total in Scopus (*n* = 1, 124) and Google Scholar (*n* = 723). A total of 1,612 records were screened by title and abstract after the removal of 235 duplicates; 1,481 were excluded at this stage. Seventy-three of the 131 full texts assessed for eligibility were excluded for the above reasons. In total, 58 studies that met all inclusion criteria were included in the final synthesis, which was organized around five thematic domains: classical ML and ensemble methods (*n* = 13), deep learning for imaging based screening (*n* = 24), explainable AI (*n* = 10), generative models (*n* = 7), and multimodal or EHR-integrated approaches (*n* = 4).

#### 3.1.1 Bibliometric characteristics of the evidence base

The systematic review was conducted in the Scopus and Google Scholar databases for the time period of January 2020–June 2026. All 58 included studies were published within this window. After duplicate removal and applying title, abstract, and full-text appraisal as per the PRISMA 2020 statement, a total of *n* = 58 studies fulfilled the pre-specified inclusion criteria. The entire evidence base extends across six thematic search clusters: (i) osteoporosis and AI, (ii) osteoporotic imaging, (iii) multimodal learning, (iv) explainable AI (XAI), (v) GANs and synthetic data generation, and (vi) diffusion models. Among the included studies, the temporal distribution is significantly biased toward more recent years, with the most publications (*n* = 47, 81.0%) being published after 2024 up to June 2026, which can be indicative of the rapid growth of interest in applying AI in research related to bone disease. The year 2026 contributed 25 studies (43.1%) alone, which signifies the up-to-dateness of the evidence integrated in this review.

As clarified in Section 2.1, the final search was completed on 30 June 2026, and the 2026-dated references included here are all peer-reviewed, published (including online-first/early-access) articles carrying a citable DOI, not preprints—consistent with the eligibility criteria in Section 2.2.

In terms of location, the evidence base is extensive but unbalanced. East and South-East Asian institutions, mainly from China, South Korea, Taiwan, and India—were the largest group (*n* = 32, 55.2%), in line with the high epidemiological burden of osteoporosis in aging Asian populations and the rapid expansion of AI research infrastructure in these countries. North American (including the United States and Canada) institutions contributed roughly 20.7% of the included studies, then European (about 15.5%), and the rest were from Middle Eastern and South American institutions. Importantly, we did not find a single study whose primary focus was on sub-Saharan African, Central Asian, or Pacific Island communities, re-emphasizing the equity gap identified in Section 4.3.

Considering the thematic breakdown between the five synthesis domains defined in Section 2.4, deep learning for opportunistic imaging-based screening remained the most common (*n* = 24 studies, 41.4%), exemplifying sustained efforts in deriving bone health metrics from radiographs, computed tomography, and magnetic resonance imaging sourced from non-bone focused clinical exams. Clinical risk prediction using classical ML and ensemble methods was the second largest group (*n* = 13, 22.4%), followed by studies on XAI integration (*n* = 10, 17.2%), applications of generative models such as GANs and diffusion models (*n* = 7, 12.1%), and by those on multimodal fusion and electronic health record (EHR) based methods (*n* = 4, 6.9%). The relative upsurge of XAI and generative model works from 2024 to 2026 appears significantly higher compared with classical ML works, indicating a more mature state of the field beyond pure predictive performance that is potentially further appropriated by invoking transparency and even data augmentation.

In terms of publication sources, the studies were distributed among various journals and conferences that were peer-reviewed. Most of the original research was published in clinical and translational journals, such as NPJ Digital Medicine, Journal of Medical Systems, BMC Medical Informatics and Decision Making, and Abdominal Radiology, reflecting an evolving clinical focus of AI-based bone disease tools. A lesser but increasing amount of work was demonstrated in quintessential AI and bioengineering publications such as the IEEE Journal of Biomedical and Health Informatics and Medical Image Analysis. Approximately 10.3% of the included studies were conference publications, mainly IEEE symposia, and were only included if they presented adequate methodological information and sample size to be eligible for inclusion.

Characteristics of the study design also influence how evidence is interpreted. Most of the included studies were retrospective in nature (*n* = 49, 84.5%), with only a handful conducted in prospective or temporal validation manners (*n* = 5, 8.6%) or reporting multicenter external validation (*n* = 4, 6.9%). The sizes of the datasets ranged widely to include cohorts as small as an *N* of 50 in exploratory MRI studies to population-based surveys of over 18,000 persons (KNHANES) and biobank repositories with over 500,000 participants (UK Biobank). The outcome label reference standard was DXA-based bone mineral density *T*-score in the vast majority of studies (*n* = 44, 75.9%), with radiological assessment-based vertebral fracture grading being the outcome in the rest. Table 1 summarizes the performance of AI techniques that were found by the primary studies in terms of a variety of relevant indicators for each imaging modality and algorithmic approach and serves as the main quantitative reference for the upcoming thematic synthesis in Sections 3.2 to Sections 3.6.

**Table 1:** Performance of different AI models for osteoporosis prediction.

Study/Model	Modality	Method	AUC	Accuracy
Wani and Arora (2024) Ensemble CNN	Knee X-ray	ResNet + AlexNet Ensemble	0.95	94.8%
Athira and Gunasudari (2025) DenseNet-CBAM	Knee X-ray	DenseNet121 + CBAM	0.97	97.43%
Athira and Gunasundari (2025) ResNet GRU	Knee X-ray	ResNet-50 + GRU	0.96	95.65%
Zhang et al. (2025) HealthiVert-GAN	CT (vertebral)	GAN + morphology modules	Binary: ≈0.95; Multi-class: ≈0.89–0.90	Binary: ≈91%–92%; Multi-class: ≈78%–81%
Padmanabha and Pai (2025) CTGAN + RF + SHAP	Clinical (tabular)	CTGAN + Random Forest + SHAP	0.89	Original dataset: 83%
Alili et al. (2026) SHIELD/NLP	Radiology reports	RadBERT RoBERTa + LLM	1.00	100%
Ramamoorthy et al. (2026) Multi-modal	Knee X-ray + metadata	2D+3D Multi-task DL	0.92	94.8%
Serpa et al. (2026) Abdominal CT	Abdominal CT	2D U-Net + ResNet34	0.96 (int)/0.82 (ext)	91%
Wang Q. et al. (2026) Stacking Ensemble	Clinical/KNHANES	Stacking Ensemble (5 learners)	0.95	96%
Zhang et al. (2026) Non-radiographic	Clinical/KNHANES	XGBoost + SHAP	0.845/0.876 (val)	72.4%
Jayarajah et al. (2026) Rho AI, X-ray	X-ray (Rho tool)	Proprietary DL	0.80–0.89	PPV 74%

int, internal validation; ext, external/multicenter validation; PPV, positive predictive value; CBAM, Convolutional Block Attention Module; GRU, Gated Recurrent Unit; NLP, natural language processing.

Table 2 provides an overview of the main data sources employed in AI-based osteoporosis studies, describing their modality, typical data set size, availability, and representative clinical applications. The comparison reveals that public repositories (KNHANES, UK Biobank) offer large-scale standardized data sets for model development, whereas most imaging research is based on small, institution-specific cohorts. Furthermore, synthetic datasets created with GAN-based methods have more and more been increasingly used to mitigate the class imbalance problem and data scarcity.

**Table 2:** Comparison of data sources used in AI osteoporosis research.

Dataset/Source type	Modality	Typical scale	Access	Representative use
KNHANES national surveys (Zhang et al., 2026; Wang Q. et al., 2026)	Clinical/Tabular	3,000–180,000	Public	Tabular BMD risk prediction
UK Biobank Xu et al., (2026)	Imaging + Clinical + Omics	>500,000	Managed access	Bone marrow adiposity, opportunistic phenotyping
Single-institution CT cohorts (Shariatnia et al., 2026; Yıldız Potter et al., 2024)	Abdominal/Spine CT	200–2,500	Restricted	Opportunistic BMD & VCF detection
Single-institution radiograph (Chen et al., 2026; Wani and Arora, 2023)	Knee/Chest/Hip X-ray	200–2,400	Restricted	Opportunistic screening, knee classification
Lumbar MRI cohorts Zhen et al., (2024)	MRI (T1/STIR/T2)	50 – 1,350	Restricted	Osteoporosis categorization from MRI
Public screening dataset (EM attenuation) Carvalho et al., (2025)	Electromagnetic signal	Labeled, open	Public	Benchmarking, radiation-free screening
Synthetic/GAN-augmented sets (Padmanabha and Pai, 2025; Zhang et al., 2025)	Tabular and Imaging	Variable	Generated	Class-imbalance mitigation

As presented in Table 2, the included studies covered a wide range of data types, from national population surveys to institutional imaging cohorts to publicly available benchmark datasets as well as to synthetic datasets derived from generative models. Public datasets, such as the KNHANES and the UK Biobank, enabled clinical and multimodal research on a large scale, and most of the studies on CT, MRI, and radiography used small institutional datasets. The proliferation of GAN-synthetic datasets is yet another testament to the increasing attempts to address the data scarcity problem and imbalanced classes, including in osteoporosis prediction. This variety in data sources exemplifies the potential and the difficulties in creating a powerful and generalizable AI system for bone disease prediction.

Figure 3 shows the theme-based classification of the 58 studies included in this systematic review. Deep learning was the largest research area (41%), which indicates its dominant role in osteoporosis diagnosis and bone disease prediction. Machine learning (23%) was the next most common study group, followed by explainable artificial intelligence (17%), which indicates increasing focus on interpretability of models. Generative models (12%) and multimodal learning approaches (7%) were emerging research lines (first-order themes), indicating further development of synthetic data generation and multimodal data fusion to enhance prediction performance.

**Figure 3:**
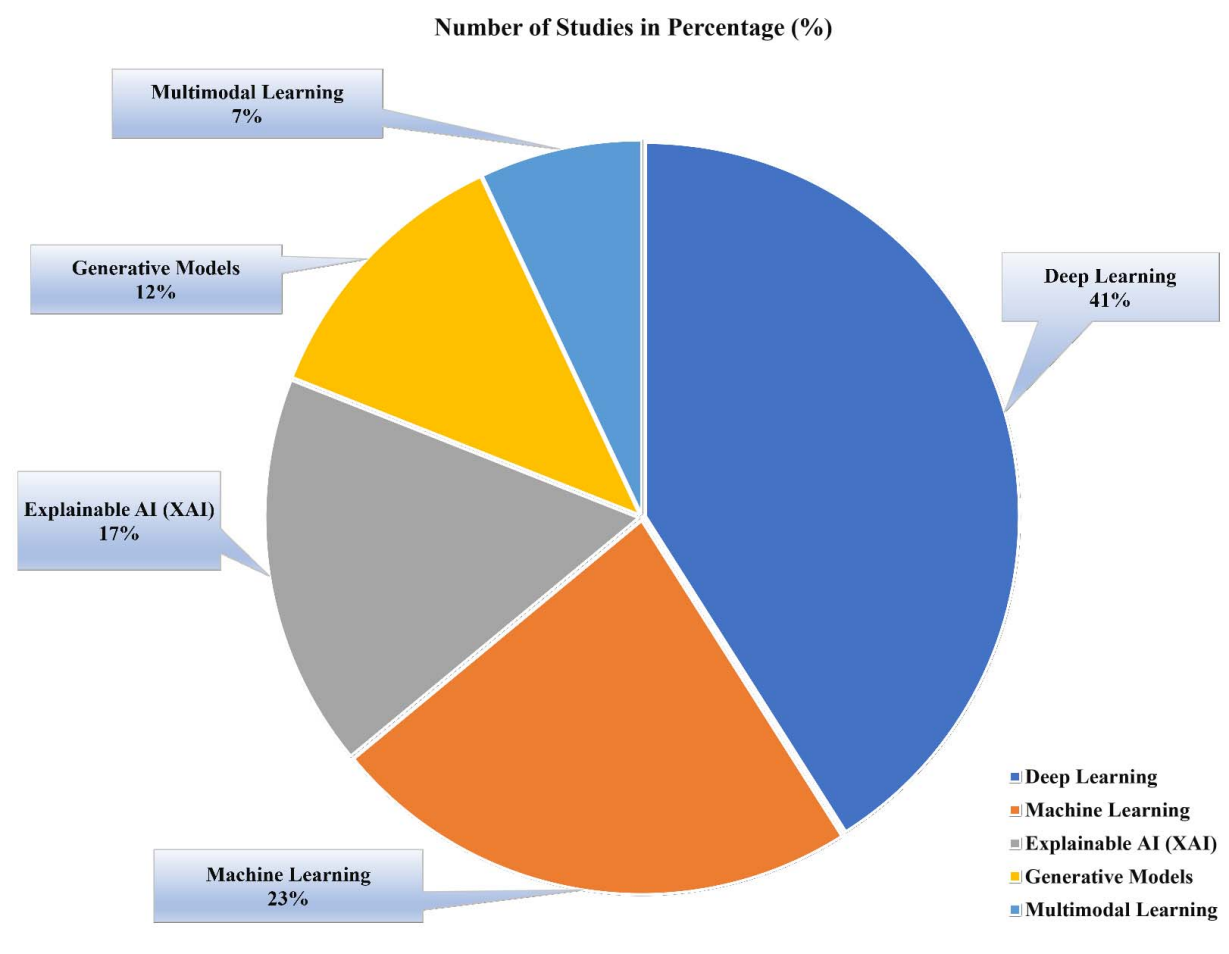
Distribution of included studies across research themes.

Figure 4 shows the temporal distribution of the studies included in the analysis. The number of publications has substantially increased in recent years, with 47 (81.0%) studies published during the period from 2024 to June 2026, while 11 (19.0%) were published in 2020–2023. This pattern reflects the rapid expansion of artificial intelligence research in osteoporosis and bone disease, especially after breakthroughs in deep learning, explainable AI, and generative models.

**Figure 4:**
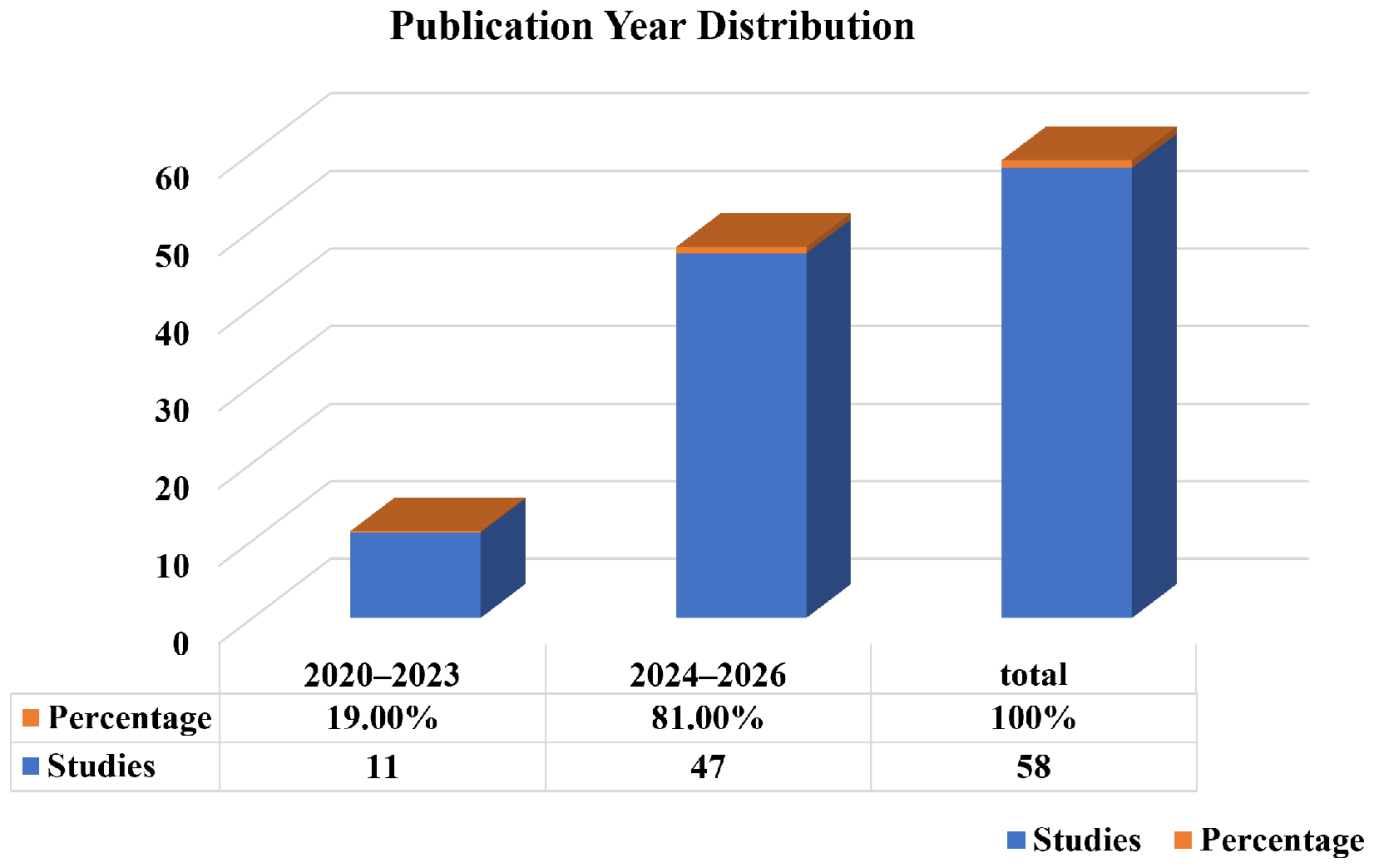
Publication year distribution of included studies.

Figure 5 displays the spatial distribution of the included studies. East and South-East Asia accounted for the space the largest share of the publications (55.2%), followed by North America (20.7%) and Europe (15.5%). Only 8.6% of the studies were from the Middle East and South America, and there were no suitable studies from sub-Saharan Africa or Pacific Island regions. These results point to a skew in geography within the current evidence and highlight the need for greater international representativeness in future osteoporosis AI research.

**Figure 5:**
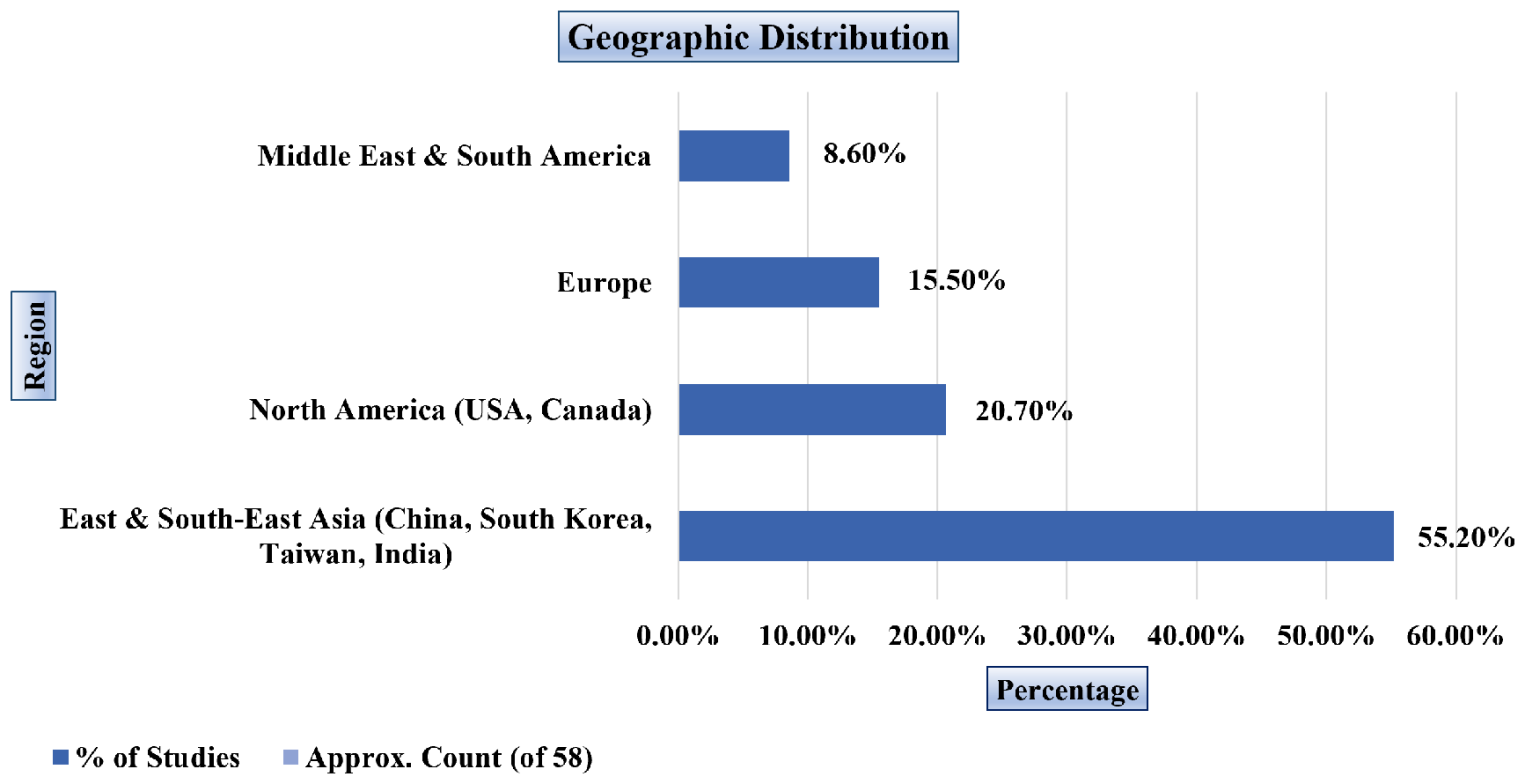
Geographic distribution of included studies.

#### 3.1.2 Quality (risk-of-bias) assessment summary

As noted in Section 2.3, a full domain-by-domain quality appraisal of the 58 included studies using PROBAST, QUADAS-2, and the Newcastle-Ottawa Scale has not yet been completed and is not claimed here; this is recorded as a limitation in Section7. In its place, this section reports study-design characteristics that were captured for every included study during data extraction and that are directly relevant to risk of bias, so that readers can form their own provisional judgement pending completion of the full instrument-based appraisal. Among the 58 studies, most (49/58, 84.5%) were retrospective, single-center studies; just 5/58 (8.6%) described prospective recruitment and 4/58 (6.9%) described external or multicentre validation (Table 1). The retrospective single-center design without external validation is usually judged as being at high risk of bias for participant selection and analysis domains by PROBAST and QUADAS-2 standards, due to convenience sampling, minimal to no management of missing data, and no pre-registered analysis plan. In contrast, prospective enrolment/ external/temporal validation are anticipated to be rated more favorably after formal assessment. The relevance probably poses in most studies since inclusion was limited to an explicit bone-health outcome and a valid reference standard was available (DXA-derived BMD or radiological fracture grading; Table 1). Rather than formal per study ratings, these design-level indicators are utilized qualitatively in Sections 4 and 5 to attenuate confidence in pooled performance figures, and are reflected in the maturity ratings presented in Figure 6.

**Figure 6:**
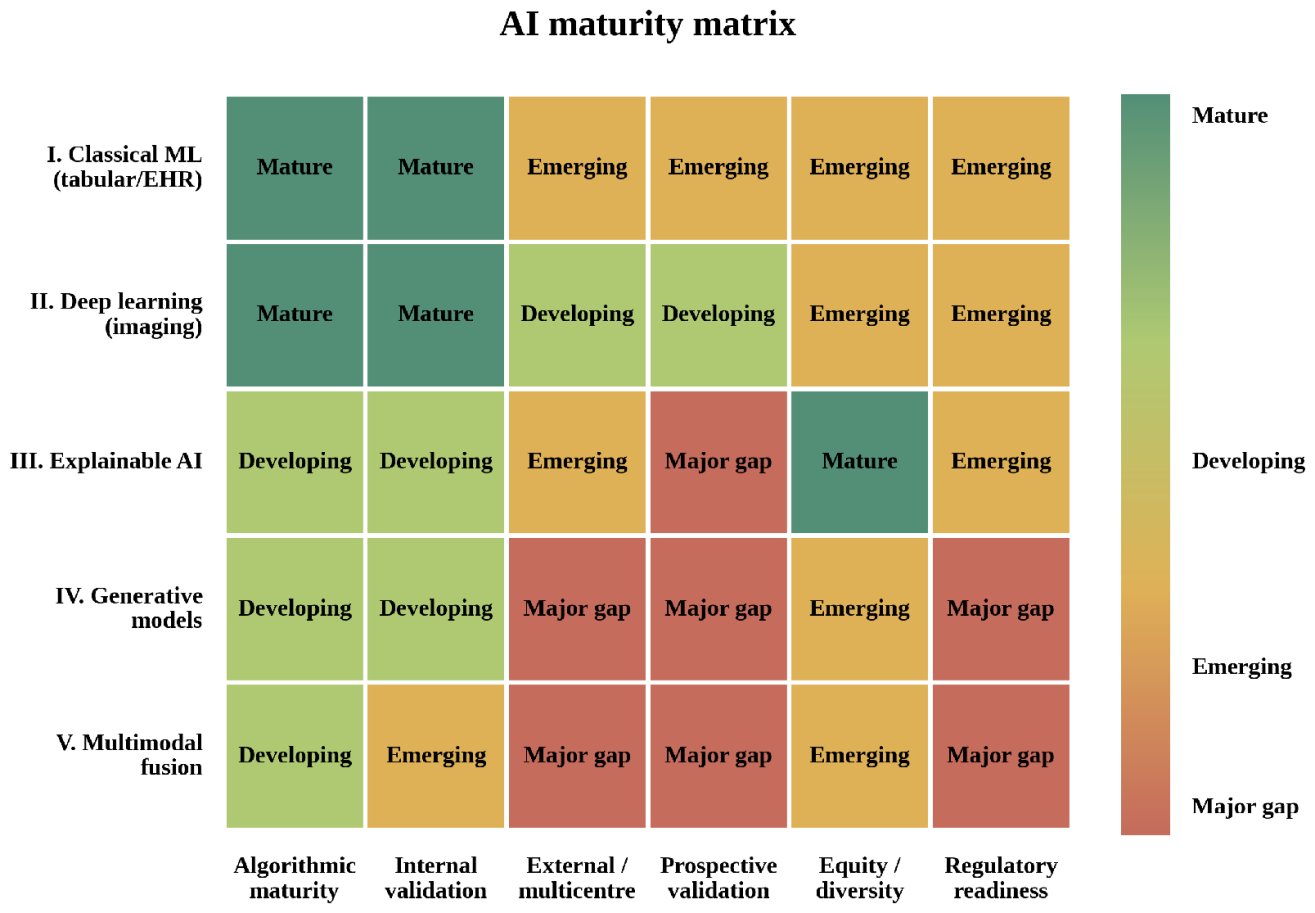
Research gap and methodological maturity matrix across AI-based osteoporosis research domains.

#### 3.1.3 Quantitative landscape summary

In addition to the theme, year, and geography level breakdowns presented in Figures 3–5, Table 3 provides a consolidated overview of the evidence landscape, summarizing the distribution of study design, validation strategy, and reference standard across the 58 included studies.

**Table 3:** Summary of study characteristics (*n* = 58).

Characteristic	Category	*n* (%)
Study design	Retrospective, single- or multi-center	49 (84.5)
	Prospective/temporal validation cohort	5 (8.6)
	Multicentre external validation reported	4 (6.9)
Thematic domain	Classical ML/ensemble (tabular, EHR)	13 (22.4)
	Deep learning for imaging-based screening	24 (41.4)
	Explainable AI (XAI) integration	10 (17.2)
	Generative models (GAN/diffusion)	7 (12.1)
	Multimodal/EHR-integrated fusion	4 (6.9)
Reference standard	DXA-based BMD/*T*-score	44 (75.9)
	Radiological vertebral fracture grading	14 (24.1)
Publication window	2020–2023	11 (19.0%)
	2024–June 2026	47 (81.0%)

Tables 4, 5 below further break down the 58 included studies by AI model/architecture category and by validation strategy, respectively, complementing the dataset (Table 3), imaging-modality (Table 4), and explainability-technique (Table 5) summaries already reported elsewhere in this review.

**Table 4:** AI model/architecture categories among 58 studies.

AI model/Architecture category	*n* (%) of 58 studies
Classical ML/ensemble methods (logistic regression, random forest, XGBoost, SVM, stacking)	13 (22.4)
CNN-based deep learning (ResNet, DenseNet, VGG, U-Net, attention/CBAM, ViT, CNN-RNN hybrids)	24 (41.4)
Explainable AI techniques applied atop classical/DL models (SHAP, Grad-CAM, LIME, attention rollout, NLP/LLM triage)	10 (17.2)
Generative models—GAN-based (CTGAN, HealthiVert-GAN, dual-GAN, autoencoder-GAN, LDCT-GAN)	7 (12.1)
Multimodal/EHR fusion architectures (joint imaging + clinical + EHR, LLM-based fusion)	4 (6.9)

**Table 5:** Validation strategies among 58 studies.

Validation strategy	*n* (%) of 58 studies
Retrospective, internal validation only (train/test split or k-fold, no external data)	49 (84.5)
Prospective or temporal validation (same institution, later time period or cohort)	5 (8.6)
External/multicentre validation (independent institution or population)	4 (6.9)

### 3.2 Machine learning for clinical risk prediction

#### 3.2.1 Ensemble methods and tabular prediction

Ensemble techniques have undoubtedly been a solid option for clinical risk stratification, providing consistently strong predictive performance. For example, Wang et al. constructed a two-level stacking ensemble based on 2017–2020 NHANES data, with 3,735 participants in the cohort. Instead of using a single algorithm, they fused five base learners: logistic regression, a decision tree, gradient boosting, XGBoost, and a multilayer perceptron, using a logistic regression-based model as the meta-learner for combining their outputs.

To provide a rigorous assessment, the group adopted stratified five-fold cross-validation, which guarded against the concern that the results were merely a consequence of a particular way the data happened to be partitioned. The stacking ensemble also achieved exceptional results in all performance metrics, reaching an AUC of 0.95, an accuracy of 0.96, a precision of 0.83, a recall of 0.81, and an F1-score of 0.82. Not only did these numbers blow all the individual base models out of the water, but even against class imbalance were surprisingly stable, with the model maintaining reassuringly low false positive rates. When they examined the variables that were most predictive, age, sex, BMI, and serum uric acid were the strongest predictors.

Zhang et al. (2026) developed a non-radiographic calculator for predicting osteoporosis based on the KNHANES data (*n* = 18, 179). Following Gradient Boost-RFE selection of features with a number of 15 key predictors, XGBoost reached high performance with AUC = 0.845 and specificity = 0.897 to predict the abnormal DXA outcome. In the three-class classification (normal/osteopenia/osteoporosis), the accuracy was 72.4% in development and 74.4% in temporal validation. The model was made available as a web-based calculator for non-invasive rapid screening. SHAP values allowed for feature-level interpretability, with age, BMI, and gender emerging as dominant predictors. This is a clinically useful advancement–a validated, free, online tool for the screening of BMD risk at the community level without the inconvenience of radiological exposure.

Machine learning has even been used in more advanced clinical cases, such as RA-associated osteoporosis. Ensemble XAI frameworks were further applied to RA patients, revealing immune-metabolic drivers of bone loss (Lin et al., 2026). Similarly, studies for identifying biomarkers applied a combination of WGCNA with LASSO and random forest feature selection to extract gene signatures relevant to osteoporosis, such as ABCA8, as a new immunoregulatory target mediated through Th17/Treg imbalance (Yi et al., 2026).

#### 3.2.2 Fracture risk prediction models

Clinical prediction models for postmenopausal osteoporosis are the subject of systematic reviews that have identified substantial between-study variability in the choice of predictors, model designs, and validation approaches (Huang et al., 2026; Li et al., 2026). Hybrid machine learning algorithms tailored for the elderly achieved better discriminative ability in predicting fractures compared to traditional FRAX-based estimations (Liu et al., 2024). Machine-learning models developed to predict the risk of refracture after vertebral compression fracture surgery also appear promising for personalized intervention, although their generalizability is limited to small sample sizes and mostly retrospective designs (Shi et al., 2026). More recently, combining oral microbiota profiles and clinical risk factors by using machine learning methods has been reported as a new strategy for osteoporosis prediction in high-altitude residents, suggesting the potential value of unconventional biological markers in improving predictive power (Wang J. et al., 2026).

*Cross-study synthesis*. The 13 classical ML/ensemble studies (Table 1) combine for three coherent rather than isolated results. In fact, stacking and boosting ensembles (Wang Q. et al., 2026; Zhang et al., 2026) achieved consistent 5–10 AUC improvements over single-algorithm baselines on NHANES/KNHANES-type tabular data, suggesting that ensembling is a relatively low-cost, high-return diversification enhancement for tabular osteoporosis risk models regardless of the specific base learners being used for the particular task. Second, age, sex/gender, and BMI were the most consistently among the top predictors in all tabular-based analyses (Wang Q. et al., 2026; Zhang et al., 2026). Although this strengthens biological plausibility, it also says that these models do not bring much more to the table than traditional clinical risk scores do; the added value of ML is primarily in calibration and modeling non-linear interactions rather than revealed predictors. Third, fracture-risk-specific models (Section 3.2.2) generally outperformed FRAX-based prediction, yet viable head-to-head comparisons within the same cohorts were based only on two single-center, retrospective studies almost in their entirety (Liu et al., 2024; Shi et al., 2026). Therefore, the above improvements over FRAX should be considered as preliminary and need confirmation in larger, prospective and externally heterogenous populations, mirroring exactly the pattern of risk-of-bias identified in Section 3.1.2.

### 3.3 Deep learning for opportunistic imaging-based screening

#### 3.3.1 Chest X-ray based opportunistic screening

Opportunistic screening, the use of an existing imaging study performed for other clinical reasons to detect unsuspected osteoporosis, has recently emerged as a transformative AI opportunity (Zheng et al., 2026). Chen et al. (2026) established an AI model in 2,384 asymptomatic Taiwanese subjects who received regular health check-ups. The model yielded an AUC of 0.95 (95% CI: 0.93–0.99) with a sensitivity of 79.7% for detecting abnormal BMD, and decision curve analysis indicated the greatest net benefit compared with “refer all” or “refer none” strategies, particularly in those with normal BMI. To our knowledge, this work is the first to promote diagnostic parity in Asian cohorts, which have been consistently under-researched in osteoporosis.

The “Rho” AI tool was prospectively assessed in 987 patients (Jayarajah et al., 2026). Rho detected 655 patients as Rho-Positive with a Positive Predictive Value of 74% for DXA *T*-score < −1. Radiologist report availability was 80%, and 78% of primary care providers were satisfied with Rho. Importantly, radiologist-reported Rho-Positive cases were also more likely to discuss bone health with their physician (37 vs. 21%, *P* < 0.001) and to be newly diagnosed with osteoporosis (12 vs. 5%, *P* = 0.02), showing measurable downstream clinical effect. These results confirm the suitability of clinical workflow integration for AI-based opportunistic screening.

The identification of mortality risk has also been associated with AI-based chest x-ray classification, extending the clinical utility of BMD from estimation to more comprehensive cardiovascular metabolic risk evaluation (Tsai et al., 2024). Foundation models combining chest CT vision language paradigms (Blankemeier et al., 2026) and AI electromyography for osteoporosis detection (Hsing et al., 2025) are frontier extensions of the opportunistic screening paradigm, with the potential to enable multi-disease screening from a single examination (Hsing et al., 2025).

#### 3.3.2 CT-based deep learning

Abdominal CT provides a particularly bountiful opportunistic screening resource due to the extent of its clinical utilization. Serpa et al. (2026) developed a ResNet34-based 2D U-Net for vertebral body segmentation as well as BMD from routine abdominal CT, with an AUC = 0.96 for internal validation and AUC = 0.82 in four external centers (*n* = 504, median age 66). The Pearson correlation for mean CT attenuation and DXA BMD was *r* = 0.62, with 100% sensitivity and 91% specificity at the optimal threshold. Multicenter validation is an essential methodological strength that differentiates this work from many single-institution reports.

Substantial progress has been made in vertebral fracture detection. Yıldız Potter et al. (2024) proposed a fully automatic vertebrae localization/segmentation and osteoporotic vertebral compression fracture (VCF) detection pipeline based on DL on CT, reaching 96% sensitivity and 81% specificity at the vertebral level, and 100% accuracy at the subject level. Of note, VCF detection improved by up to 14% in sensitivity and 20% in specificity when using predicted vertebra segments as model inputs, which shows the potential benefit of segmentation-first architectures. AI has been similarly utilized to detect vertebrae that need to be excluded from lumbar DXA analysis because of the presence of artifacts, thus increasing measurement precision (Sebro et al., 2023).

Comparisons of AI-assisted BMD estimation with Quantitative CT (QCT) in elderly populations demonstrated high agreement (Guo et al., 2025), indicating that AI has the potential to produce QCT-equivalent results from non-specialized scans. Vertebral-level discrimination based on BMD texture features and FE-derived vertebral load is a high-level multi-feature approach connecting imaging biomarkers to biomechanical risk (Strack et al., 2026).

#### 3.3.3 Knee X-ray deep learning for osteoporosis

Knee X-rays are among the most frequently obtained radiographs worldwide, and the potential for opportunistic screening is high. Several DL models were tested. DenseNet-121 with Convolutional Block Attention Module (CBAM) was proposed by Athira and Gunasudari (2025) for knee X-ray classification, resulting in an accuracy of 97.43% by integrating channel-wise and spatial attention to enhance feature representation and mitigate the problem of vanishing gradients. The same group's ResNet-50 + Gated Recurrent Unit (GRU) hybrid (Athira and Gunasundari, 2025) obtained a 95.65% accuracy by integrating spatial feature extraction and temporal dependency modeling, which highlights the benefit of incorporating recurrent architectures into even static imaging tasks when supplemented with sequential clinical information.

Wani and Arora (2024) studied ensemble DL for knee X-ray osteoporosis detection in a Kashmir Valley population (*n* = 240) and reported that ensemble schemes based on DNN with AlexNet and ResNet were best among the considered cases. Integration of clinical covariances (age, sex, and BMI) with imaging CNN features led to additional improvement in discrimination. Ramamoorthy et al. (2026) combined 2D X-ray analysis with 3D bone density reconstruction and FRAX-based fracture risk calculation with an AUC = 0.92 and accuracy = 94.8%, forming a multi-task learning system for clinical use. Transfer learning-based CNN methods for knee X-ray diagnosis were shown to have similar performance while requiring substantially less training data (Wani and Arora, 2023).

VGG16 with a logistic regression layer on radiograph and BMD data also obtained correct osteoporosis classification (Salunke et al., 2025). Deep learning for the classification of bone quality using DXA images was reportedly useful in automating morphological evaluation (Gonzalez et al., 2024). A hierarchical multi-layer opportunistic screening approach based on clinical data and CT images in a conventional three-layer architecture was proposed to deliver a more refined risk stratification pipeline (Liu et al., 2022).

#### 3.3.4 Cross-modality comparison

The four imaging modalities used for AI-based bone analysis differ significantly in cost, availability, radiation dose, and proven efficacy, as outlined in Table 6. Chest and knee radiographs have the greatest availability and lowest cost and have been well-suited to opportunistic deep-learning screening, while CT offers volumetric, QCT-like data at the expense of a greater dose. MRI, long underutilized for bone because of weak cortical signal, is now demonstrated to enable osteoporosis classification from routine lumbar T1/STIR/T2 sequences using custom CNNs (Nemane et al., 2026), paving a radiation-free volumetric path. DXA is still the diagnostic gold standard, but it is the least amenable to opportunistic, scalable use.

**Table 6:** Comparison of imaging modalities for AI-based osteoporosis assessment.

Modality	Availability/Cost	Radiation	AI tasks demonstrated	Reported performance	Key limitations
DXA (Sebro et al., 2023; Gonzalez et al., 2024)	Moderate	Low	Bone-quality classification, artifact exclusion	High concordance	Limited portability; reference standard, not opportunistic
Chest X-ray (Chen et al., 2026; Tsai et al., 2024)	High/Low	Low	Opportunistic BMD classification, mortality risk	AUC 0.95 (CI 0.93–0.99)	2D projection; view dependence
Knee X-ray (Athira and Gunasudari, 2025; Ramachandran and Charles, 2025; Sarhan et al., 2024)	High/Low	Low	Osteoporosis classification, multi-task fusion	Accuracy 95%–97%	Site-specific; small cohorts
Abdominal/spine CT (Shariatnia et al., 2026; Yıldız Potter et al., 2024; Guo et al., 2025)	Moderate/High	Higher	BMD estimation, VCF detection, QCT-equivalent	AUC 0.82–0.96	Dose; specialized acquisition
MRI (lumbar) (Mousavinasab et al., 2025)	Lower/High	None	Osteoporosis categorization from T1/STIR/T2	Custom CNN, exploratory	Cost; weak cortical signal; small data

*Cross-study synthesis*. Amongst 24 deep-learning studies based on imaging, a pattern is consistent: internal-validation performance is reported as high and with a relatively small variance (accuracy for radiograph based classification ranges from 91 to 97%; AUC for CT and X-ray based screening ranges from 0.92 to 0.97), but seldomly is this performance challenged outside the development cohort. Of all the CT-based studies, Serpa et al. (2026) was the only one to report multicenter external validation, and it demonstrated an approximately 0.14 AUC decline when compared to internal validation (Section 3.7)—the largest drop, single best direct evidence in this review that internally validated imaging-DL performance is optimistic when compared to real-world implementation. A second general pattern is modality-driven tradeoffs, not a singular “best modality”: chest and knee radiographs have the highest availability–performance ratio for population-based opportunistic screening; CT provides the closest approximation to QCT-equivalent quantitative BMD, albeit with a dose and cost penalty; and MRI is considered experimental, with the smallest and least externally validated evidence base of the four modalities (Table 6). 3rd- Architecture choice (CNN vs. attention-augmented CNN vs. hybrid CNN-RNN) yielded relatively small (2%–5% point) accuracy differences on exactly the same knee-X-ray task (Athira and Gunasudari, 2025; Wani and Arora, 2024), implying that dataset size/quality and validation rigor are probably more important for real-world application of this task than additional architectural advances.

### 3.4 Explainable AI for bone disease interpretability

#### 3.4.1 The interpretability imperative

The application of black-box AI models in the clinical management of bone disease poses fundamental issues of trust, accountability, and regulatory adherence. Clinicians need more than accurate predictions; they need actionable explanations: which features contributed to this patient's fracture risk assessment? Why has this vertebra been highlighted? What is the mechanistic meaning of this X-ray pattern? XAI techniques try to provide answers to these questions by presenting *post-hoc* or ante-hoc (inherently interpretable) explanations of models' (Alili et al., 2026) behaviors.

Three main XAI paradigms have been used for the prediction in osteoporosis and bone disorder are: (1) SHapley Additive exPlanations (SHAP), which computes a contribution score based on the principles of game theory for every feature; (2) Gradient-weighted Class Activation Mapping (Grad-CAM), which generates visual heatmaps emphasizing image areas that lead to DL classification; and (3) Local Interpretable Model-agnostic Explanations (LIME), which replaces complicated models locally with interpretable ones. All have unique strengths for different modalities of data (tabular vs. imaging) and clinical communication applications.

#### 3.4.2 SHAP for tabular bone disease prediction

Padmanabha and Pai (2025) oresented an XAI solution based on a CTGAN synthetic data generation space with Random Forest classification and SHAP interpretation for modeling the risk of osteoporosis. Employing CTGAN to address the problem of limited data in clinical settings, the proposed model demonstrated outstanding results on augmented data, while SHAP values indicated age, BMI, and history of previous fractures as the main risk contributors. To the best of our knowledge, this is the first work to offer a methodological template for the dual challenge of data scarcity and interpretability in osteoporosis AI.

The non-radiographic prediction calculator in Zhang et al. (2026) also used SHAP values to explain XGBoost predictions and created patient-level feature contribution plots that could be viewed by clinicians, as well as the predicted risk score. This design represents an important step toward “clinician-in-the-loop” AI implementation, in which model output is combined with interpretable reasoning. Gradient-based feature selection (GradientBoost-RFE) with LASSO regression retained only the most clinically relevant predictors, avoiding dilution of SHAP explanations due to redundant features.

ML origin for osteoporosis biomarker identification from transcriptomic data using LASSO, random forest, and Boruta feature selection coupled with GSEA/ssGSEA enrichment analysis (Yi et al., 2026), yielding gene-level explanations linking molecular signatures to immune dysregulation in osteoporosis. Interpretable ML-based postmenopausal osteoporosis risk prediction has been deployed as non-radiographic online calculators promoting AI-enabled bone health evaluation to clinicians around the world (Zhang et al., 2026), advancing the accessibility of AI-driven bone health assessment.

#### 3.4.3 Grad-CAM and visual explanation for imaging

Skeletal Health Intelligence and Early Lesion Detection (SHIELD) (Alili et al., 2026) is an advanced XAI application combining RadBERT-RoBERTa NLP for radiology report triage with large language model (LLM) generated natural-language explanations. Fine-tuned on 10 years of radiology reports (*n* = 245 patients), SHIELD achieved perfect accuracy and an area under the curve (AUC) of 1.00 for binary referral classification, with 90% accuracy for three-class triage (No Referral/Referral/Referral-High Risk). The LLM explanation component produced a clinician-interpretable rationale, which enabled auditability. Importantly, the system did not confuse a high-risk situation as one that did not require referral (“fail-safe” clinical conduct), and shortened median referral time from 109.6 days to 1–3 min.

For image-based osteoporosis evaluation, Grad-CAM visualizations on bone-quality classifiers have shown correct focus on trabecular texture patterns and cortical boundary regions, consistent with radiological expertise. Several studies (Li et al., 2026; Liu et al., 2024) and DenseNet-CBAM-based models (Athira and Gunasudari, 2025) confirmed that the model concentrated on the subchondral bone region and cortical-trabecular interface—clinically important regions—rather than incidental background features. In vertebral-fracture detection, Yıldız Potter et al. (2024) used Grad-CAM to verify that their DNN models focused on endplate integrity and vertebral height ratios, as expected clinically.

Importantly, XAI techniques have exposed model failure modes not visible in aggregate metrics. In some knee X-ray DL models, Grad-CAM revealed a focus on soft-tissue shadow rather than bone texture, i.e., features opposite to domain expectations, despite acceptable aggregate accuracy. Such “negative” XAI findings are arguably more valuable clinically than performance numbers alone, since they help avoid deploying models that are misleadingly accurate but mechanistically invalid.

#### 3.4.4 Comparison of explainability methods

The explainability methods used in the reviewed works cater to different data types and clinical communication levels and vary in their fidelity, stability, and computational cost in Table 7. SHAP dominates Excel in tabular prediction for bone disease due to the game-theoretic property of its feature attributions; Grad-CAM and attention-based visualizations dominate in imaging; and natural-language report generation (RadBERT/LLM pipelines like SHIELD) is beginning to appear as a layer of explanation for the clinician (Alili et al., 2026). None of the included articles measured explanation faithfulness or stability to perturbation, highlighting the disunity in Section 4.1.

**Table 7:** Comparison of explainable-AI methods in bone-disease models.

XAI method	Data type	Mechanism	Output	Representative use	Limitations
SHAP (Padmanabha and Pai, 2025; Zhang et al., 2025)	Tabular	Game-theoretic Shapley values	Per-feature contribution	XGBoost/RF risk calculators	Cost on large feature sets; assumes feature independence
Grad-CAM (Yıldız Potter et al., 2024; Athira and Gunasudari, 2025)	Imaging	Gradient weighted activation	Saliency heatmap	CNN attention verification	Coarse localization; class discriminative only
LIME (Alili et al., 2026)	Tabular/Imaging	Local surrogate models	Local feature weights	Model-agnostic explanation	Instability across runs; local-only
Attention rollout (Athira and Gunasudari, 2025)	Transformer	Attention aggregation	Token/region importance	ViT and hybrid models	Attention ≠ explanation caveat
NLP/LLM report (Alili et al., 2026)	Text (reports)	RadBERT + LLM generation	Natural language rationale	SHIELD triage	Hallucination risk; needs auditing

*Cross-study synthesis*. Considered together, the 10 XAI-focused studies (and the many other studies that use XAI as a secondary component) show a clear modality-driven split: SHAP is used almost exclusively downstream of tabular/ensemble models, while Grad-CAM and attention-based maps are used almost exclusively downstream of CNN/transformer imaging models, with very little cross-over or head-to-head comparison of methods on the same task. This is itself a gap: because no included study benchmarked two or more XAI methods against each other on the same osteoporosis dataset, or quantified explanation stability/faithfulness (Table 7), the field currently has no evidence base for choosing one explanation method over another beyond convenience and data type. The recurring qualitative finding that Grad-CAM attention over-relies on soft-tissue shadow rather than trabecular bone in some knee-X-ray models is a rare and clinically important exception, and argues for XAI to be used routinely as a model-debugging step, not only as a *post-hoc* communication aid, consistent with the performance–interpretability trade-off discussed in Section 4.1.

### 3.5 Generative models: GANs and diffusion models

#### 3.5.1 GANs for osteoporosis data augmentation

The application of Generative Adversarial Networks to bone diseases has been done through two main ways: on one side (1) in synthetic tabular data generation to tackle class imbalance and data availability issues, and on the other side (2) in medical image synthesis to produce missing modalities or to amplify imaging datasets. The key issue that motivates the use of GANs in bone disease investigation is the extreme class imbalance in osteoporosis populations; only about 10%–20% of screened subjects receive a diagnosis, which greatly skews traditional ML models toward the majority class.

Conditional Tabular GAN (CTGAN) has also been used for modeling osteoporosis risk (Padmanabha and Pai, 2025), synthesizing tabular patient records with statistically realistic distribution while maintaining the correlation between age, sex, BMI, hormonal status, and BMD values. The Random Forest models trained on the CTGAN enhanced data sets showed a markedly better sensitivity for the osteoporosis class while maintaining the specificity—an important clinical trade off, sensitivity, specificity, where you want to miss as few true positive cases, i.e., fracture risk patients. SHAP analysis on enhanced data sets also validated that the synthetic data did not contaminate spurious feature relationships, and hence reaffirms the biological plausibility of the generative model.

Gu et al. (2023) proposed BMD estimation from plain X-ray images by a GAN-based decomposition, which learns projections of bone-segmented CT. Learning based on a small CT-radiograph paired dataset and exploiting the strength of GAN in modeling high-dimensional complex imaging physics, the method found great success in BMD prediction using low-cost and universally available radiographs, making it possible to perform BMD measurement wherever radiographs are obtained.

#### 3.5.2 HealthiVert-GAN: a landmark in vertebral synthesis

HealthiVert-GAN (Zhang et al., 2025) is the most advanced application of generative modeling for vertebral bone disease identified in this review. It addresses the critical clinical challenge of osteoporotic vertebral compression fracture (OVCF) grading, which normally requires comparison with pre-fracture CT scans that are seldom available, by producing pseudo-healthy vertebral images that approximate the pre-fracture state of the fractured vertebra. The approach uses a coarse-to-fine synthesis network and three auxiliary modules that exploit morphology and height information from neighboring healthy vertebrae to preserve anatomical coherence. The new RHLFV metric introduced by HealthiVert-GAN allows objective measurement of fracture severity by quantifying dissimilarity between the generated pseudo-healthy vertebra and the real fractured vertebra, converting a previously subjective radiological grading task into a reproducible, quantifiable measure. Verified with Shanghai Sixth People's Hospital data following ethics approval, the model achieved higher fracture-grading sensitivity than DL models without the synthesis step, illustrating a paradigm shift in which generative models function as clinical measurement tools rather than purely as data-augmentation tools.

#### 3.5.3 GAN architectures for medical image quality enhancement

GANs have the potential to be transformative for medical image synthesis and bone imaging pipelines in particular, beyond just bone-specific applications. Dual-GAN-based (Seg2GAN) (Beji et al., 2023) yielded 8%–50% gains in Dice scores for segmentation by producing plausible synthetic training samples. Autoencoder-based GAN frameworks (Cao et al., 2023) facilitate multimodal image generation from partial data—such as in osteoporosis scenarios where patients may own a CT but no MRI, or vice versa. Variation-driven neural networks (Chen et al., 2024) that explicitly model the data distribution lead to better cross-modality synthesis (*CT*↔*MRI*) and allow bone density information to be derived from modalities obtained for other reasons. Noise-encoding GAN-based LDCT image synthesis (Li et al., 2023) allows the determination of BMD from low-radiation protocols, taking into consideration patient safety in the screening applications. GAN-based stochastic image model evaluation (Kelkar et al., 2022) found that standard metrics (FID, IS) do not necessarily capture dimensions of image quality important for medical images, leading to the proposal of domain-specific GAN evaluation frameworks for applications in bone imaging.

#### 3.5.4 Diffusion models: the emerging paradigm

GAN-based approaches dominated medical image synthesis in 2022–2023; from 2024 onward, diffusion models have emerged as a more advanced paradigm addressing GAN limitations such as training instability, mode collapse, and limited diversity. Prompt-guided synthetic data generation with diffusion models has mitigated domain shift in periapical-lesion detection, a scheme directly transferable to vertebral-fracture and BMD evaluation ('´Oz'´ud'´oğru et al., 2026). Stable-diffusion-based frameworks for multiclass lesion segmentation have also shown competitive results on CT-based tasks ('´Ozcan, 2026).

A review of generative AI for synthetic data in healthcare (Waseem et al., 2026) concluded that diffusion models have reached parity with, or outperformed, GANs in image fidelity, diversity, and clinical realism. SADiff, a sinogram-aware diffusion model for LDCT denoising (Niknejad Mazandarani et al., 2025), showed that physics-informed diffusion models surpass GAN-based solutions for CT image-quality enhancement. The nascent area of StyleGAN-based path analysis (Zhang, 2025) and CIPHER cross-modal diffusion models (Wu et al., 2026) provides avenues for multimodal bone-imaging synthesis and translation.

In the case of osteoporosis applications, diffusion models offer potential theoretical benefits such as better mode coverage to capture the variability of bone microarchitecture, generation of higher-quality and more diverse synthetic samples, potential text-conditioning to synthesize images with specific fracture patterns and/or BMD ranges, an iterative refinement mechanism that can be naturally embedded in multi-scale bone structural analyses, and better sample efficiency to reduce the number of training images. However, forward validation studies applying diffusion generation specifically to osteoporotic bone and BMD enhancement are currently lacking, a gap this review highlights as a priority.

#### 3.5.5 GANs vs. diffusion models: a comparative appraisal

The shift from GAN-based to diffusion-based medical image generation is among the most striking methodological changes in the body of literature. Table 8 compares the two paradigms very well in the two most relevant dimensions for bone imaging. GANs remain compelling for their fast, single-pass sampling and have produced the field's landmark clinical tool in HealthiVert-GAN (Zhang et al., 2025); diffusion models, while more computationally intense at inference, have superior mode coverage, training stability, and conditioning flexibility, and are increasingly popular for high-fidelity synthesis and domain-shift mitigation (Niknejad Mazandarani et al., 2025). As is the case in osteoporosis, comparative evidence is still substantially borrowed from neighboring domains, and toe-to-toe assessment on bone tasks is a priority gap.

**Table 8:** GANs vs. diffusion models for bone-imaging synthesis.

Dimension	GANs	Diffusion models
Training stability	Prone to instability, mode collapse	Stable; likelihood-based objective
Sample diversity/mode coverage	Limited	High
Inference speed	Fast (single pass)	Slow (iterative denoising)
Conditioning flexibility	Moderate (cGAN, CTGAN)	High (text/mask/class)
Data efficiency	Moderate	Improving, still data-hungry
Bone-disease examples	CTGAN, HealthiVert-GAN	Largely transferred from adjacent domains
Best current use	Pseudo-healthy synthesis, tabular augmentation	High-fidelity synthesis, domain-shift mitigation

*Cross-study synthesis*. Across the seven generative-modeling studies, two trends stand out. First, there is a clear architectural transition underway (GAN-dominant through 2023, diffusion-dominant from 2024), but this transition is far more mature in adjacent radiology domains (chest, brain, and general oncologic imaging) than in bone-specific applications, where HealthiVert-GAN remains essentially the only bone-specific generative model with rigorous clinical validation (Section 3.5.2); most of the diffusion-model evidence cited here is extrapolated from non-bone applications (Section 3.5.4). Second, and consistent with the equity concerns raised in Section 4.3, none of the generative studies reported whether synthetic samples were validated separately in underrepresented subgroups (e.g., men, non-Asian ethnicities, atypical fracture patterns); validation of GAN/diffusion output has so far focused on aggregate downstream classifier accuracy rather than on the realism or fairness of the generated samples themselves, which is the central methodological gap taken up in Section 4.2.

### 3.6 Multimodal fusion and EHR integration

The merging of imaging, clinical information, laboratory results, and -omics data provides the richest foundation for precision osteoporosis treatment. Liu et al. (2022) have designed a hierarchical three-layer opportunistic screening model combining clinical risk factors with CT-based imaging features, showing that integrated models significantly outperformed either modality alone. This hierarchical design first screens by clinical risk, then identifies high-risk subgroups, offering a cost-effective, scalable implementation.

EHR integration has been proven to be an accelerator for osteoporosis AI. Almog et al. (2020) showed that EMR-DXA integration facilitates reporting, eliminates errors, and enables intelligent downstream analysis. Representation learning of EHR data using autoencoders. Deep representation learning of EHR data (Landi et al., 2020). A deep learning-based autoencoder mechanism has become a popular approach. This leads to patient stratification, which predicts clinically distinct osteoporosis subtypes associated with comorbidities, medication history, and disease trajectory. The KESER approach (Hong et al., 2021), facilitating clinical knowledge extraction through large-scale EHR embedding networks was able to detect osteoporosis-related code networks from multi-institution data without the need to share patient-level data–an important privacy-preserving solution for cross-institution work.

Large language models (LLMs) have shown promise in making electronic health record (EHR)-based predictive models more generalizable across healthcare systems. The GRASP framework (Kirchler et al., 2026) utilizes LLM-based embeddings with transformer-based prediction models and can be transferred across different countries for predicting disease onset in a case-control study involving more than a million individuals. This method can potentially be applied to the prediction of osteoporosis, where existing models usually suffer from severe degradation in performance when applied to populations different from that of model construction. Additionally, multimodal fusion techniques for prognostic prediction from other cancers (Tian et al., 2025) also provide a methodological template to effectively integrate imaging, genomic, and clinical data for predictive modeling of bone.

Foundation models are the cutting-edge in multimodal bone disease AI (Blankemeier et al., 2026). A CT vision-language foundation model with a large-scale radiology dataset shows the possibility of models which are at the same time, understand anatomical context, clinical description, and quantitative measurements, allowing a single model to perform BMD estimation and fracture detection as well as report generation for a wide range of clinical presentations.

Table 9 provides a summary of representative multimodal and EHR-integrated methodologies, describing the modalities, the fusion technique, and the main contributions of the method.

**Table 9:** Fusion approaches combining clinical and imaging modalities.

Approach	Modalities fused	Fusion strategy	Principal advance
Hierarchical screening (Liu et al., 2022)	Clinical + CT	Sequential (clinical then imaging)	Cost-effective triage pipeline
EHR-based deep learning risk prediction (Almog et al., 2020)	EHR (longitudinal ICD codes)	Sequential embedding + LSTM/XGBoost	Improved short-term fracture-risk AUROC (0.67 → 0.82)
Deep EHR representation (Landi et al., 2020)	EHR (longitudinal)	Convolutional autoencoder	Unsupervised patient subtyping
KESER (Hong et al., 2021)	Multi-institution EHR embeddings	Sparse embedding regression	Privacy-preserving knowledge extraction
GRASP/LLM transfer (Kirchler et al., 2026)	EHR + LLM embeddings	Transformer + LLM features	Cross-country transferability
Merlin Foundation Model (Blankemeier et al., 2026)	CT + language	Vision-language pretraining	Unified multi-task representation
Multi-task knee model (Ramamoorthy et al., 2026)	X-ray + metadata + FRAX	Joint 2D/3D multitask	Imaging + clinical risk in one model

*Cross-study synthesis*. The four multimodal/EHR studies and three related foundation-model/LLM-transfer works (Table 9) converge on a single message: fusing even one additional data type with imaging consistently improved discrimination over single-modality baselines (Liu et al., 2022; Ramamoorthy et al., 2026), yet multimodal fusion is simultaneously the least mature of the five thematic domains in this review by every readiness dimension in Figure 6—algorithmic maturity, internal validation, and especially external/prospective validation and regulatory readiness. This combination (consistent performance gains, but the smallest and least externally validated evidence base of the five themes) makes multimodal fusion the domain with the largest gap between demonstrated potential and current clinical readiness, and is the central motivation for the MXGNet roadmap proposed in Section 6.

### 3.7 Quantitative synthesis of reported performance

Before presenting the pooled figures below, we reiterate the caveat introduced in Section 2.5: the AUC values compared in this section come from studies with different sample sizes, populations, reference standards, and critically different validation protocols (internal-only vs. external/temporal). They are reported here as a descriptive summary of the published evidence, not as a formal meta-analytic comparison, and should not be read as demonstrating that any single modality or model is definitively superior to another.

Among the 58 identified studies, eight also reported discrimination as an area under the receiver-operating characteristic curve (AUC) in a way that was directly comparable across studies; these are the quantitative subset synthesized here. Another category of knee radiograph deep learning works (Wani and Arora, 2024, 2023) had a single osteoporosis vs. normal AUC reported were accuracy (usually 91%–97%) or AUCs per class; such works are not discussed in the pooled descriptive statistics, but in a narrative manner instead. Since there were significant differences in the definition of outcomes, reference standards, and validation methodologies between studies, pooling by inverse-variance was not performed (Section 2.5); the following represent a descriptive summary of published point estimates rather than meta-analytic effects. Reported internal-validation AUCs were skewed toward the higher end, with a median of 0.94 (interquartile range, 0.86–0.95) across modalities (Figure 7).

**Figure 7:**
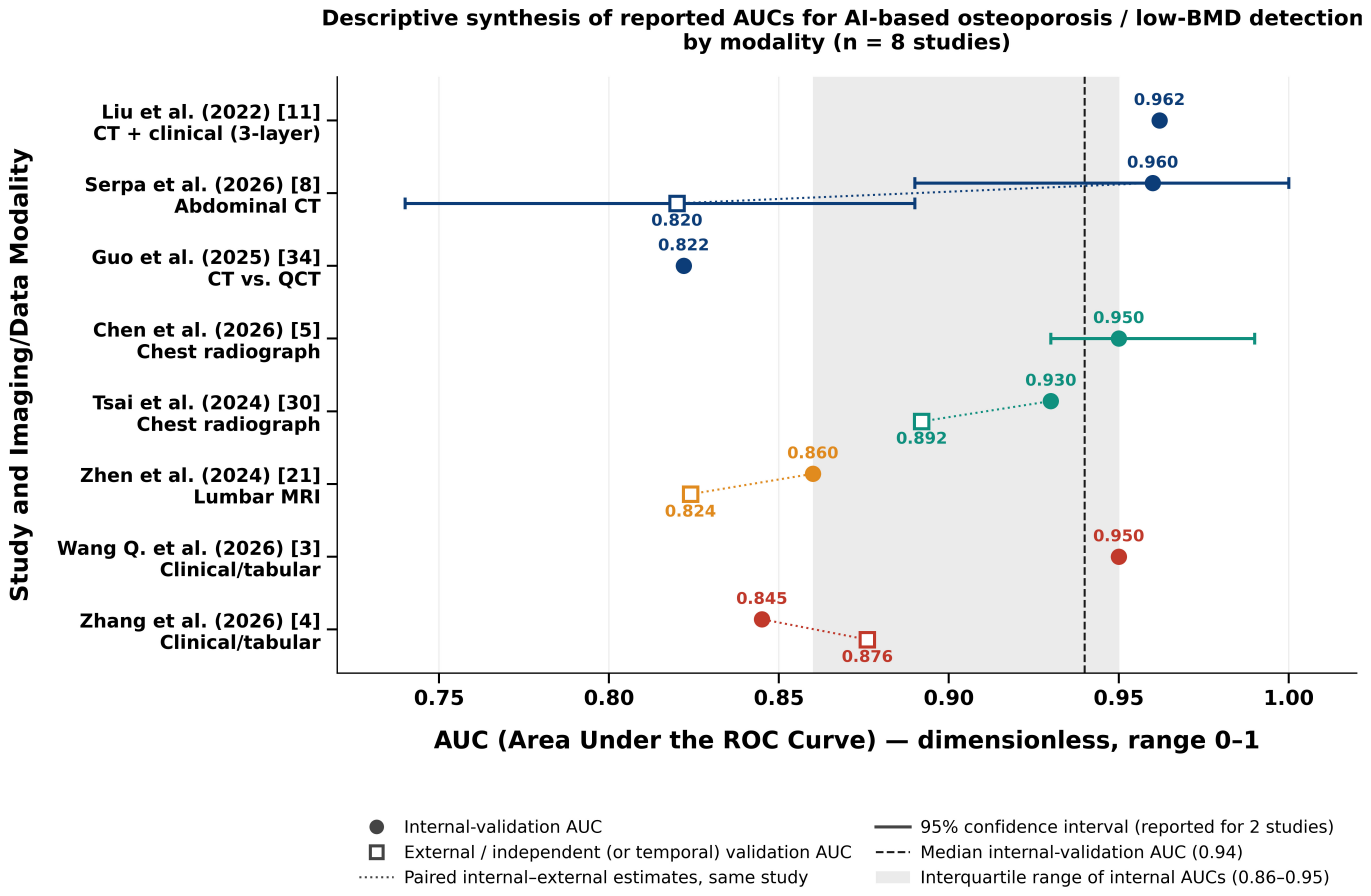
Descriptive synthesis of reported AUCs for AI-based osteoporosis/low-BMD detection, by modality (*n* = 8 studies).

The highest internal-validation performance was reported for opportunistic computed-tomography-based methods: Liu et al. (2022)'s hierarchical clinical and CT model reported AUC = 0.962, and Serpa et al. (2026)'s multicentre abdominal-CT framework achieved AUC = 0.96 (95% CI: 0.89–1.00) on internal validation. Chest-radiograph-based screening also performed well: Chen et al. (2026) reported a population-level AUC = 0.95 (95% CI: 0.93–0.99), and Tsai et al. (2024) obtained an internal-validation AUC of 0.93. Zhen et al. (2024) developed a multiparametric lumbar MRI radiomics model with an internal AUC of 0.86, and Guo et al. (2025) obtained AUC = 0.82 in a CT- and QCT-based study.

The most informative quantitative signal in the entire pipeline is not any single summary value but the steady decrease in performance on out-of-sample or external test sets. Where both were available, the abdominal-CT pipeline dropped from 0.96 internally to 0.82 (95% CI 0.74–0.89) on external data a decrease of about 0.14 in AUC (Serpa et al., 2026); the chest-radiograph classifier dropped from 0.93 to 0.89 (Tsai et al., 2024); and the lumbar-MRI model dropped from 0.86 to 0.82 on external institutional data (Zhen et al., 2024). One tabular model behaved differently, slightly improving on temporal validation (0.845 → 0.876) (Zhang et al., 2026), consistent with the lower ceiling but higher stability sometimes observed for low-dimensional clinical predictors. The effect-size degradation on external validation is therefore real and, at its largest, clinically meaningful (~0.14 AUC), but variable rather than a constant offset it should moderate enthusiasm about very high values reported under internal validation alone.

Models developed with structured clinical data were also well-competitive in performance with imaging. The stacking-ensemble classifier proposed by Wang Q. et al. (2026) attained the test-set AUC of 0.95 (accuracy 0.96), while the non-radiographic interpretable calculator of Zhang et al. (2026) achieved AUCs of 0.845 (internal) and 0.876 (temporal validation). These results support the observation made elsewhere in this review that interpretable, low-cost predictors can equal or exceed complex deep learning systems for some screening problems, while being far easier to deploy and audit. Values reported as near-perfect discrimination from small samples were treated with caution and excluded from the descriptive synthesis, as likely small-sample or task-specific artifacts.

Discriminative performance of studies with directly comparable AUC values (*n* = 8; Table 10). Values were extracted from the original publications, with 95% confidence intervals included when available. Knee radiograph studies reporting only accuracy or class-specific AUCs are summarized in the text and were excluded from this comparison.

**Table 10:** Reported internal and external AUCs across studies.

Study	Modality	Internal AUC	External/Independent AUC
Liu et al. (2022)	CT + clinical (3-layer)	0.962	–
Serpa et al. (2026)	Abdominal CT	0.96 (0.89–1.00)	0.82 (0.74–0.89)
(Guo et al. 2025)	CT vs. QCT	0.822	–
Chen et al. (2026)	Chest radiograph	0.95 (0.93–0.99)	–
(Tsai et al. 2024)	Chest radiograph	0.930	0.892
Zhen et al. (2024)	Lumbar MRI	0.860	0.824
Wang Q. et al. (2026)	Clinical/tabular	0.95	–
Zhang et al. (2026)	Clinical/tabular	0.845	0.876 (temporal)

Descriptive summary of reported AUCs for AI-based osteoporosis and low-BMD detection across imaging modalities (*n* = 8 studies; Figure 7). Filled circles represent internal validation results, while open squares indicate external or independent validation results; dotted lines connect paired estimates from the same study. Horizontal error bars denote 95% confidence intervals for the two studies that reported them (Chen et al., 2026; Serpa et al., 2026). The dashed line marks the median internal-validation AUC (0.94), and the shaded region represents the interquartile range (0.86–0.95).

A formal inverse-variance meta-analysis was not conducted because of substantial heterogeneity in outcome definitions, reference standards, and validation approaches across studies. There are three methodological limitations that need to be taken into account when interpreting the present results. First, we were unable to formally pool results due to heterogeneity in osteoporosis definitions, DXA reference populations, and validation approaches; thus, the median and inter quartile range present a measure of central tendency describing the distribution of reported AUCs rather than a pooled estimate. Second, only two of the eight studies provided 95% confidence intervals for their primary AUC estimates (Chen et al., 2026; Serpa et al., 2026). This means that it was not possible to formally quantify between-study heterogeneity (I-squared) and publication bias. Third, the majority of the studies were retrospective, and external validation of results with independent datasets was performed in three out of eight comparable studies (Serpa et al., 2026; Zhen et al., 2024; Tsai et al., 2024). Thus, the reported performance may be an upper bound when generalizing to real-world scenarios. Futures studies are also warranted to adhere to TRIPOD+AI promises and incorporate uniform external validation among diverse populations and imaging modalities to allow for trustworthy quantitative meta-analyses.

## 4 Novel analytical insights

### 4.1 The performance—interpretability spectrum

One of the main consequences of this systematic review is the apparent inverse association between the predictiveness and explainability of the AI algorithms designed for bone disease applications. The highest-performing models, in particular deep CNNs and transformer-based models, generally achieve maximum performance with AUC values around 0.90–0.96, but the decision logic is hardly interpretable. In contrast, traditional machine learning techniques, such as logistic regression and decision trees, are inherently interpretable but generally achieve weaker predictive performance with AUC values around 0.75–0.87. Explainable ensemble algorithms, like stacking models with SHAP as explainability, appear to provide a satisfactory balance between these two objectives. These models have also been found to generate explanatory outputs that are more intuitive and clinically meaningful to healthcare providers and perform better than other explainable techniques with AUCs in the range of 0.90–0.95.

As emphasized throughout Section 3 (and consistent with the caution given in Section 3.7), these AUC ranges are drawn from studies using different populations and validation protocols and should be read as an approximate, descriptive pattern rather than a precise, directly comparable ranking.

This review also underlines a key consideration that goes beyond the conventional trade-off between predictive performance and interpretability: the readiness of AI models for clinical and regulatory use. Although black-box models with high predictive performance tend to excel in terms of predictive accuracy, their opaqueness poses challenges to gaining approval and adoption in the healthcare domain, as regulatory agencies are putting more emphasis on explainability. Models that utilize explainable AI methodologies, on the other hand, may be more suitable for clinical translation. Still, a key challenge that remains is addressing how reliable and consistent the explanations that they produce are. For instance, the robustness and reliability of SHAP based feature attribution have not yet been sufficiently studied in rare bone diseases. Future work should therefore focus on developing common assessment protocols for explainable AI in this area, moving away from the mere reporting of the use of explanation methods toward the assessment of quality, robustness, and clinical validity of the produced explanations.

### 4.2 The data scarcity generativity paradox

Generative models are commonly advertised as a solution to the problem of data scarcity, but the development of successful models is usually reliant on having access to sufficiently large and diverse datasets. This obstacle applies especially to these populations underrepresented in osteoporosis research: men, children, and those with extreme bone loss accompanied by atypical fracture patterns. In these scenarios, the limited availability of training samples may also constrain the ability of GANs and diffusion models to synthesize biologically valid samples. Therefore, a more feasible strategy may be to transfer the knowledge from a large-scale medical image dataset and then apply the pre-trained models to the small bone disease datasets by fine-tuning.

The other major takeaway from this review is that there is little advancement in the validation of synthetic datasets for bone disease. The available works mainly aim at proving the increase of the model's accuracy after augmentation with GANs, but the evidence about the medical realism of synthetic bone images is still very limited. In particular, remains to be seen to what extent they truly capture clinically important texture patterns, and whether any model trained on synthetic data using silicon itself runs and performs well in varied real-world conditions. Clarification of these questions by means of standardized evaluation would be a prerequisite for clinical application of bone disease AI based on synthetic data.

### 4.3 Equity gap analysis

This review also highlights a recurring lack of patient representation within bone-disease AI studies. Of the 58 studies included, the vast majority focused on postmenopausal women, although osteoporosis also affects men, appears in children with inherited and acquired disorders of bone fragility, and differs significantly between ethnic groups, for which the bone geometry and bone mineral density reference values are not uniform. And a little more positive representation for the Asian community, with the likes of Chen et al. (2026) and studies based on the Korean National Health and Nutrition Survey (KNHANES); however, minorities of African, South Asian, and Latin American descent remain grossly under-represented. This disparity has direct clinical outcomes. Risk prediction models constructed largely from Caucasian women may systematically underestimate fracture risk in other populations, which questions their utility and suggests an urgent need for equitable, safe implementation.

A related issue is the generalizability of imaging-based models between healthcare systems with different amounts of resources. Algorithms trained on high-resolution DXA or CT data from resource-rich institutions should not be assumed to generalize to lower-resolution images obtained from resource-limited institutions. This limitation is apparent even in ideal circumstances: the X-ray screening device tested by Jayarajah et al. (2026) in Canadian hospitals, where the availability of imaging technology is much “higher,” still failed to detect a significant number of cases. The performance of such models in day-to-day, real-life practice is, to a large extent, unknown. The research leading to these results has therefore focused on ensuring that future work includes geographically and socioeconomically diverse populations and uses equity-centered evaluation metrics, so that performance can be assessed not only “overall” but also for the specific subpopulations that will ultimately be the tool's stakeholders.

### 4.4 Research-gap and maturity matrix

Figure 6 summarizes the results of this review, in the levels of methodological maturity for thematic domains based on seven dimensions of readiness, from algorithm development to regulatory preparedness. This is not a surprise, and the figure shows a common pattern that is sometimes obscured when studies are taken on a one by one basis. Except for a few areas, such as external and prospective validation, inclusion of diverse populations, and regulatory readiness, most disciplines present significant advances in algorithm development and internal validation. These limitations are even more pronounced for generative and multi-modal methods, where a significantly smaller number of external validations have been performed, and only a handful of different populations have been tested. In contrast, as inherently designed to enhance model transparency, explainable AI methods are implemented to be more transparent and to offer explanations about the behavior of AI in the clinical decision-making process, it is unknown if these explanations affect clinical decision-making or patient outcomes. Thus, the matrix is to be read as a qualitative integration of the existing literature and is not a quantitative evaluation, and informs the future research priorities that are outlined in Section 6.

Figure 6 shows the matrix of research gap and maturity across the five thematic domains across the seven Readiness dimensions. Cells are the result of a qualitative assessment synthesized over the included studies. The cells represent the authors' qualitative assessment of methodological maturity synthesized from the included studies, rather than a quantitative scoring system. The most prominent gaps, indicated by red and amber regions, are concentrated in external and prospective validation, equity representation, and regulatory preparedness, particularly for generative and multimodal approaches.

The cells in Figure 6 represent the authors' qualitative synthesis of the included studies, informed by the risk-of-bias appraisal described in Section 2.3 and summarized in Section 3.1.2, rather than a quantitative scoring system. The most prominent gaps, concentrated in external/prospective validation, equity representation, and regulatory preparedness, are most pronounced for generative and multimodal approaches—directly motivating the MXGNet roadmap presented in Section 6.

## 5 Discussion

This review combines the findings of 58 studies to summarize the current knowledge on AI, explainable deep learning (XAI), and generative models in bone and osteoporosis prediction.

First, opportunistic screening based on AI represents a paradigm shift in osteoporosis care. Using imaging data already obtained for other reasons such as chest X-rays, abdominal CT scans, and even ECGs AI can identify individuals at risk at no extra cost, radiation, or inconvenience to patients. The evidence presented shows AUCs ranging from 0.82 to 0.96 for DL based BMD estimation using opportunistic imaging (Table 10), indicating clinical-grade performance in some scenarios, while also illustrating the internal-to-external validation gap discussed in Section 3.7. The remaining challenge is therefore less about algorithm development than about deployment, integration into clinical workflows and PACS pipelines, and validation across varied populations and imaging equipment.

Second, explainability is transitioning from being an academic curiosity to a clinical and regulatory necessity. The SHIELD framework and the HealthiVert-GAN's objective height-loss metric are examples of how XAI principles can be embedded in clinical solutions rather than applied after the fact, as is the case with LLM-generated explanations. The best of what the future holds, for work, is going to be in explicability as a first-order end, not as an afterthought. What constitutes “enough” explanation for a range of clinical decisions will require insights for AI engineers, clinicians, patients, and regulators.

Third, the generative models are evolving into a real clinical tool from a data generator tool. The ability of HealthiVert-GAN to generate pre-fractured vertebral states for objective grading and the CTGAN to generate clinically plausible tabular patient data are indications that generative models can create data that do not exist in the real world, but are clinically necessary for a sufficient evaluation. In light of the clear superiority of diffusion models over GANs in visual quality and diversity, the bone diseases community is encouraged to rapidly follow suit and apply these architectures for the task of low-dose CT enhancement, missing modality synthesis, and rare subpopulation augmentation.

Finally, multimodal data fusion is essential for the next generation of osteoporosis research and care. Although unimodal AI models demonstrate good predictive power, they are unlikely to capture the full complexity of bone health. The combination of dual-energy X-ray absorptiometry images with electronic health record data (including medication history and corticosteroid and bisphosphonate use), laboratory data (including vitamin D, parathyroid hormone, and calcium), omics-derived biomarkers, and patient-reported outcomes may lead to more individualized approaches for assessment of osteoporosis risk and development of care plans. In this sense, vision-language foundation models may provide a unifying general-purpose framework to integrate various data modalities and generate personalized osteoporosis management strategies significantly outpacing the traditional population-based risk stratification.

Several methodological limitations in the reviewed literature, and consequently in this review, suggest that the results above should be interpreted with caution. To make the evidentiary limitations underlying the review's conclusions explicit: the great majority of primary studies (*n* = 49, 84.5%; Section 3.1.1) were retrospective, which may introduce selection bias and limits causal inference. Only a small minority (*n* = 4, 6.9%) conducted external validation with independent, multicentre data, meaning the generalizability and clinical readiness of most reported models remains unconfirmed; the ~0.14 AUC drop observed when external validation was performed (Section 3.7) suggests that internally reported performance is frequently optimistic. Dataset sizes varied widely, from small exploratory cohorts to population-scale biobanks, so models built on the smallest cohorts are likely especially prone to overfitting even where internal cross-validation was used. Publication bias is also a plausible concern: because negative or null AI results are much less likely to be submitted or accepted for publication than positive ones, the true distribution of model performance in this field is probably less favorable than the published record in Table 8 and Figure 7 suggests, and this review being based only on published, peer-reviewed literature (Section 2.1) cannot correct for that bias. Finally, substantial heterogeneity in study design, osteoporosis definitions, reference standards, imaging-acquisition protocols, and DXA hardware made formal meta-analysis infeasible (Section 3.7). Taken together, these factors mean the high internal-validation AUCs reported throughout Section 3 should be regarded as best-case, not typical-case, evidence of real-world diagnostic performance.

Upcoming studies should be prospective and multi-center, with pre-specified primary outcomes, should undergo independent external validation, and should adhere to standardized reporting guidelines such as the TRIPOD+AI recommendations to enhance methodological quality, reproducibility, and clinical real-world translation.

## 6 Future research roadmap

Based on the findings of the present systematic review, six research priorities are identified to support the continued development of artificial intelligence for bone disease applications during the next 5 years. Section 6.1 presents MXGNet as a conceptual framework intended to address several methodological and translational challenges highlighted by the reviewed studies.

The results consolidated from this review indicate that various significant challenges in bone disease AI, such as data scarcity, single modality, lack of model transparency, and limited generalizability on different populations, are not sufficiently mitigated by the existing solutions. To offer a possible avenue for future research, is to suggest a conceptual framework, entitled Multimodal eXplainable Generative Network (MXGNet), based on the three major concepts emerging from this survey: generative data augmentation, MM data integration, and intrinsically explainable modeling. MXGNet is not designed to be a validated predictive model but rather a template for new research in the next generation of AI systems for predicting bone disease. A block diagram of the proposed framework is shown in Figure 8.

**Figure 8:**
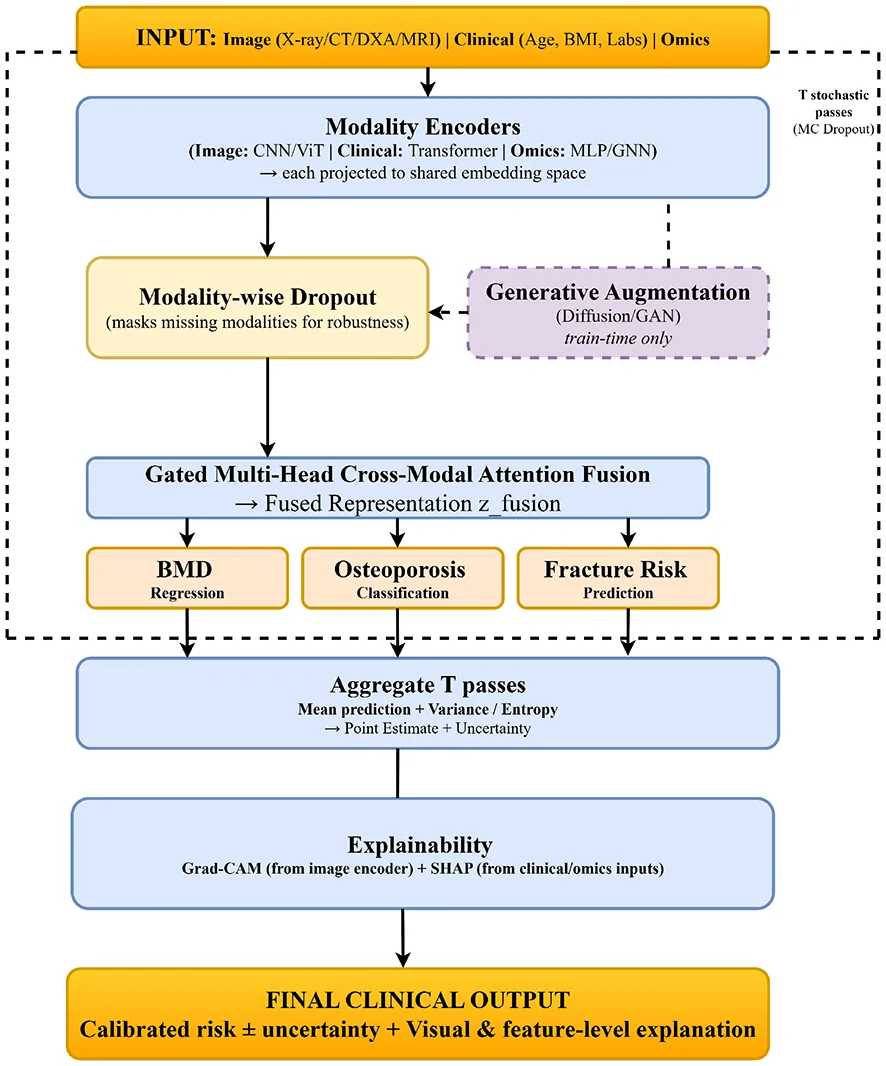
MXGNet—Proposed Multimodal eXplainable generative network for bone-disease prediction.

Our proposed Multimodal eXplainable Generative Network. Encoder specific to the modality are input into a gated cross-modal attention fusion core, augmented by a generative branch to cope with data scarcity, with intrinsic SHAP/Grad-CAM explainability and uncertainty-aware prediction heads.

### 6.1 Architecture overview

MXGNet runs in these four nested stages. Within the first stage, encoders specific to modality convert the heterogeneous sources of data into a unified latent embedding. Imaging information from X-rays, CTs, DXA, or MRIs is processed with a convolutional neural network or vision transformer to produce an image embedding, represented as *z*_*img*_. Clinical and electronic health record data, such as demographic information, laboratory results, and medication histories, are processed via a tabular transformer to produce a clinical embedding, *z*_*clin*_. In a similar way, omics profiles and biomarker information are encoded as an embedding, *z*_*omic*_, utilizing either a multilayer perceptron or a graph neural network. Stage two adds a generative augmentation module based on diffusion or GAN algorithms, as the HealthiVert framework is inspired. This portion also operates as a generator for realistic samples for minority classes and rare patient subpopulations to help address issues of data scarcity and class imbalances. In the third stage, the embeddings of each modality for a given entity are combined at the entity level with a gated cross-modal attention mechanism, and modality dropout is adopted to improve robustness against missing modalities. Also, our final layer prediction heads can provide uncertainty estimates of predicted bone mineral density, osteoporosis status, and fracture risk. To improve model transparency, the framework adds inherent explainability functionality, SHAP, to explain tabular features, and Grad-CAM to highlight informative areas in medical images.

### 6.2 Mathematical formulation

Three modality-specific encoders, parameterized by learnable weights, produce latent representations for imaging, clinical, and omics data. These embeddings are then fused by a gated multi-head cross-attention mechanism whose gated scalar weights and learnable attention weights encode the importance of each modality in the fused representation. In order to enhance the resilience against incomplete data, the Bernoulli-based modality dropout technique is integrated into the training, enabling the framework to sustain the prediction performance even though one or more modalities are missing in the testing. Afterwards, task-specific prediction heads process the fused representation to predict bone mineral density, osteoporosis status, and fracture risk. Then, epistemic uncertainty is estimated through Monte Carlo dropout by taking the average over the predictions from multiple stochastic forward passes.

Model learning is guided by a combined loss function that takes into account supervised learning, generative augmentation, representation alignment, and prediction calibration. Focal cross-entropy loss is applied to deal with the class imbalance that is frequently seen in osteoporosis cohorts, where the minority classes rarely exceed 10%–20% of the total population under study. The generative augmentation branch is trained with a diffusion- or adversarial-based loss, and the progressive augmentation is optimized with Huber regression loss to predict DXA-derived bone mineral density. To incentivize well-calibrated probabilities, an expected calibration error penalty is added, and a contrastive learning objective is introduced to encourage paired multimodal representations to be aligned in the common latent space.

To enable a formal description of the proposed MXGNet, the framework is designed as a multimodal learning model considering imaging, clinical, and electronic health record (EHR) data with gated cross-attention fusion while adopting generative learning and explainability. Let the three input modalities be denoted as


$$\begin{array}{l} X=\left\{X_{\text{img}},X_{\text{cli}},X_{\text{ehr}}\right\} \end{array}$$ (1)

In Equation 1, *X*_*img*_ denotes the medical image (such as an X-ray, CT, or MRI), *X*_*cli*_ represents structured clinical variables including age, sex, BMI, and laboratory results, and *X*_*ehr*_ is longitudinal electronic health record data.

A modality-specific encoder is utilized to separately encode each modality and acquire latent feature representations,


$$\begin{array}{l} \begin{array}{cc} z_{\text{img}} & =E_{\text{img}}\left(X_{\text{img}}\right),\\ z_{\text{cli}} & =E_{\text{cli}}\left(X_{\text{cli}}\right),\\ z_{\text{ehr}} & =E_{\text{ehr}}\left(X_{\text{ehr}}\right) \end{array} \end{array}$$ (2)

In Equation 2 *E*_*img*_, *E*_*cli*_, and *E*_*ehr*_ denotes the image encoder, clinical encoder, and EHR encoder, respectively.

To facilitate adaptive fusion of heterogeneous information, MXGNet utilizes gated cross-modal attention. For each modality, a learnable gating coefficient is calculated as


$$\begin{array}{l} \alpha_{i}=\sigma (W_{g}z_{i}+b_{g}) \end{array}$$ (3)

In Equation 3 σ(·) denotes the sigmoid activation function, *W*_*g*_ and *b*_*g*_ are learnable parameters, and α_*i*_∈[0, 1] controls the contribution of the *i*^th^ modality.

The fused multimodal representation is obtained using attention-weighted aggregation,


$$\begin{array}{l} z_{\text{fusion}}=\overset{M}{\underset{i=1}{\sum }}\alpha_{i}A_{i}z_{i} \end{array}$$ (4)

In Equation 4, *A*_*i*_ is the cross-attention weight for the corresponding modality, *M* = 3. To enhance the robustness to missing clinical data, a Bernoulli modality-dropout is applied during training


$$\begin{array}{l} {\tilde{z}}_{i}=m_{i}z_{i},\;m_{i}\sim \text{Bernoulli}\left(p\right) \end{array}$$ (5)

In Equation 5 *m*_*i*_ is a binary masking variable with the retention probability *p*. As a result, the model is still capable of making predictive inferences in the absence of any modality during test time. The prediction part is designed for joint osteoporosis risk classification and bone mineral density (BMD) estimation. The classification output is defined as shown in Equation 6


$$\hat{y}=\text{Softmax}(W_{c}z_{\text{fusion}}+b_{c})$$ (6)

while continuous BMD prediction is computed as shown in Equation 7


$$B\hat{M}D=W_{r}z_{fusion}+b_{r}$$ (7)

where *W*_*c*_, *W*_*r*_, *b*_*c*_, and *b*_*r*_ are trainable parameters.

To quantify predictive confidence, epistemic uncertainty is estimated using Monte Carlo Dropout over multiple stochastic forward passes,


$$\begin{array}{l} ŷ=\frac{1}{T}\overset{T}{\underset{t=1}{\sum }}f^{\left(t\right)}\left(X\right) \end{array}$$ (8)

In Equation 8 *T* denotes the number of stochastic inference passes, and *f*^(*t*)^(·) represents the prediction obtained during the *t*^th^ pass.

The entire learning objective is a supervised generative learning with calibration and multimodal representation learning,


$$\begin{array}{l} L=\lambda_{1}L_{\text{cls}}+\lambda_{2}L_{\text{bmd}}+\lambda_{3}L_{\text{gen}}+\lambda_{4}L_{\text{cal}}+\lambda_{5}L_{\text{con}} \end{array}$$ (9)

In Equation 9 *L*_cls_ is the focal cross-entropy loss for osteoporosis classification, *L*_bmd_ is the Huber regression loss for BMD estimation, *L*_gen_ represents the diffusion or adversarial loss related to synthetic data generation, *L*_cal_ is a calibration loss that makes the predicted probabilities more reliable, and *L*_con_ is a contrastive loss that brings representations from different modalities closer in a common latent space. The coefficients λ_1_, …, λ_5_ control the relative importance of the objectives during optimization.

The model parameters are learned by minimizing the composite objective, as shown in Equation 10


$$\begin{array}{l} \theta^{*}=\arg\underset{\theta }{\min}L\left(\theta \right) \end{array}$$ (10)

In Equation 10 θ represents all the parameters that can be trained in MXGNet. This formulation allows a consistent mathematical representation of multimodal feature extraction, adaptive attention-based fusion, uncertainty-aware prediction, and explainable learning modes, and it provides a principled basis for framing the proposed framework as well as for its realization in clinical practice.

#### 6.2.1 Explainability and workflow

In MXGNet, the explanation is added as part of the prediction process and not post-model development, as elaborated in Section 4.1. The framework outputs a calibrated risk prediction and an associated uncertainty measure per patient during inference. For clinical ease of interpretation, the model also outputs Grad-CAM maps that emphasize the image patches that most strongly influence the prediction, as well as SHAP-based feature rankings that assess the impact of clinical and omics features. These outputs are combined into a structured report designed to inform clinical decisions, akin to the reporting paradigm used by the SHIELD framework. The presented workflow aims at enabling opportunistic screening: routinely acquired medical images for unrelated clinical indications and accessible electronic health record data are analyzed collectively by MXGNet. To ensure patient safety and enable dependable decisions, the framework includes an abstention rule that suggests human evaluation once predictive uncertainty is above a certain threshold, which in turn decreases the possibility of blindly generated but erroneous predictions.

#### 6.2.2 Validation plan and equity safeguards

The MXGNet evaluation design is heavily slanted toward external prospective validation. The protocol foresees a centralized model building (federated learning) to facilitate data sharing while protecting patient privacy (Nemane et al., 2026). Model accuracy would be validated in prospective study designs using DXA based measures as the gold standard. Furthermore, it is obvious that sex, ethnicity, age, and body mass index related subgroup analysis is going to be of great importance for testing model fairness and performance homogeneity. Synthetic data augmentation can also be strategically applied to representation learning practice to mitigate the patient group under-representation concern raised in Section 4.3. Notably, MXGNet, as a hypothesis-driven platform for research and its core assumption that the combination of generative modeling, multimodal learning, and explainable AI leads to better model generalizability and thus fairer outcome predictions when compared to paradigms that would include just one methodological aspect, is subject to rigorous scrutiny in subsequent empirical studies. These may include, among others, scheduled randomized controlled trials comparing standard vs. AI-enhanced osteoporosis screening workflows in varied populations, with pre-specified equity subgroup analyses by sex, ethnicity, age, and BMI.

Application of Diffusion Model in Bone Imaging: systematic assessment of diffusion models for synthesis of osteoporotic bone, low-dose CT enhancement, and BMD-based image generation, based on standardized biological fidelity metrics.

Standardizing and Regulatory frameworks in XAI: osteoporosis case study, certain XAI assessment measures, such as explanation faithfulness, stability over perturbations, and clinical actionability scoring. Partnership with FDA/EMA on AI-as-Medical-Device pathways for XAI bone health tools.

Foundation Models for Unified Bone Disease Assessment large-scale pretraining of vision-language foundation models on heterogeneous bone image datasets (X-ray, CT, DXA, MRI, and ultrasound) to obtain unified representations that can be leveraged by various downstream tasks using a single model.

Global fairness in AI tools for bone health: focused research on African, South Asian, Latin American, and male groups; adjustment of current models for application to various ethnic reference groups; and creation of AI tools suitable for low-resource environments tested with devices commonly found in the healthcare systems of LMIC.

Federated Learning and Non-intrusive Multi-Lender AI: federated learning frameworks are implemented to allow multi-institution model training without sharing any patient information, solving the matter of contention that is multi-institution validation vs. patient privacy.

## 7 Limitations

There are various limitations to this review. First, the fast development of AI research could lead to the omission of some studies published between submission and publication. Second, diverse definitions of outcomes did not allow for meta-analysis of pooled performance. Third, there is probable publication bias leading to overestimated model performance, since negative AI studies are barely published. Fourth, since we concentrated on articles in English, we potentially missed out on some relevant research being conducted in non-English speaking countries, most notably those in Asia, where the study of osteoporosis is gaining momentum. Fifth, the review was based on study-reported metrics without re-analysis of original data, which precludes evaluation of actual model generalizability.

The following limitations of the underlying evidence base are highlighted explicitly, as they bear directly on how confidently the conclusions of this review can be generalized to clinical practice:

- Predominance of retrospective studies. 84.5% of included studies (49/58) were retrospective (Section 3.1.1), which increases susceptibility to selection bias, hindsight-informed feature engineering, and optimistic performance estimates relative to prospective clinical use.
- Limited external validation. Only 6.9% of studies (4/58) reported multicentre external validation, and where both internal and external AUCs were available, performance dropped by up to ~0.14 AUC (Section 3.7). Reported diagnostic accuracy in this literature should therefore be regarded as a best-case rather than a representative estimate of real-world performance.
- Potential publication bias. As this review draws exclusively on published, peer-reviewed literature (Section 2.1), studies reporting null or negative findings for AI-based osteoporosis screening are likely under-represented, which would tend to inflate the apparent maturity and performance of the field relative to its true state.
- Lack of prospective, multicentre validation. Prospective validation was reported in only 8.6% of studies (5/58). Combined with the limited external validation above, this means very few of the models reviewed have been tested under conditions resembling real-world, multi-site clinical deployment, and the generalizability and clinical adoption of the reported findings should accordingly be regarded as provisional pending further prospective, multicentre evidence.
- No formal, instrument-based risk-of-bias appraisal completed. A domain-by-domain quality appraisal of all 58 included studies using PROBAST, QUADAS-2, and the Newcastle-Ottawa Scale, as would be expected of a rigorous systematic review, has not been completed for this version of the manuscript (Section 2.3). Section 3.1.2 reports study-design-level indicators (retrospective vs. prospective design, validation strategy, reference standard) as a transparent proxy in its place. Completing the full instrument-based appraisal—ideally independently by both authors, with disagreements resolved by discussion—is identified as a priority next step before the review's conclusions are relied upon for clinical or policy decisions.
- Inter-reviewer agreement not quantified. Screening decisions were not logged in a form that permits retrospective calculation of Cohen's Kappa (Section 2.2), so no quantitative concordance statistic is reported for this review; agreement was instead managed through discussion-based consensus at both screening stages.
- No pre-registered review protocol. This review was not prospectively registered (e.g., with PROSPERO) prior to conduct, which is a further limitation to the methodological transparency of the review process itself, alongside the points above regarding the incomplete risk-of-bias appraisal and unquantified inter-reviewer agreement.

## 8 Conclusion

Artificial intelligence has become a game changer in osteoporosis and bone disease management, reaching clinical-grade performance in various screening, risk prediction, and fracture evaluation applications. Deep learning models obtain consistently good results with AUC 0.82–0.96 for BMD estimation through opportunistic imaging, ensemble approaches elucidated by SHAP result in clinically useful risk scores in the absence of DXA, and generative models–most notably GANs and the new diffusion paradigms–address the lack of data while creating the possibility for novel measurement paradigms such as pseudo-healthy vertebral synthesis.

Three paradigm shifts are clear from this synthesis: (1) the transition from screening as a separate clinical visit to opportunistic detection integrated into routine care; (2) the progression from performance-only assessment to performance-plus-interpretability as the application standard; and (3) the evolution of generative AI from data augmentation tools to clinical measurement tools in their own right. The convergence of these shifts, along with multimodal foundation models that combine imaging, EHR, and -omics data, positions AI to fundamentally reshape bone disease care.

However, as detailed in Sections 5 and 7, these gains are demonstrated predominantly in retrospective, internally validated studies; prospective validation, equity representation, development of regulatory frameworks, and standardized evaluation of XAI remain key barriers that must be overcome before AI bone-health tools can reach their full potential in routine clinical practice. This review proposes a concrete research roadmap (Section 6) to help close that gap. With concerted investment across the clinical, engineering, and policy domains, AI-driven management of bone disease may help narrow the worldwide osteoporosis care gap and prevent millions of avoidable fractures each year.
